# Cross-cultural adaptation and validation of a self-reporting tool to assess health-related quality of life for Egyptians with extremity bone sarcomas in childhood or adolescence

**DOI:** 10.1186/s12955-023-02165-3

**Published:** 2023-07-29

**Authors:** Nesma Farid, Sungsoo Chun, Omneya Hassanain, Mohamed Salama, Elham Esam, Fatima Adel, Ismail Rashad, Ahmed Mohamed El Ghoneimy

**Affiliations:** 1 Institute of Global Health and Human Ecology, American University in Cairo and Clinical Research Department, Children’s Cancer Hospital Egypt (CCHE-57357), Cairo, Egypt; 2 Institute of Global Health and Human Ecology, American University in Cairo, Cairo, Egypt; 3 Clinical Research Department, Children’s Cancer Hospital Egypt (CCHE-57357), Cairo, Egypt; 4 Nursing department, Children’s Cancer Hospital Egypt (CCHE-57357), Cairo, Egypt; 5 Department of Orthopedic Oncology, Cairo University and Children’s Cancer Hospital Egypt (CCHE-57357), Cairo, Egypt

**Keywords:** Egyptian, Childhood bone sarcoma, Extremity, TESS, pTESS, Patient-reported, Health-related quality of life

## Abstract

**Background:**

Validated self-reporting tools are required to evaluate the functional outcome and health-related quality of life (HRQOL) for those who had extremity bone sarcomas in their childhood or adolescence. Our study pursued cross-cultural adaptation and validation of the pediatric Toronto Extremity Salvage Score (pTESS) and Toronto Extremity Salvage Score (TESS) to assess the functional outcome for Egyptian children and adult survivors following surgeries of extremity bone sarcomas. In the modified versions of pTESS and TESS, mental domains were added to allow the evaluation of HRQOL using a specific instrument for childhood bone cancer.

**Methods:**

The internal consistency and test–retest reliability of the studied forms were assessed with Cronbach’s alpha and Intra-class coefficients (ICC), respectively. For convergent validity, correlations between scores of the generic Pediatric Quality of Life Inventory (PedsQL 4.0) and pTESS /TESS scores were reported. Factor Analysis was feasible for pTESS-leg; due to the insufficient samples, only the average inter-item correlation coefficients were reported for the remaining versions.

**Results:**

Out of 233 participants, 134 responded to pTESS-leg, 53 to TESS-leg, 36 to pTESS-arm, and only 10 to TESS-arm. All versions showed excellent internal consistency (Cronbach’s alpha >0.9), good test–retest reliability (ICC >0.8), moderate to strong correlations with PedsQL, and acceptable average inter-item correlation coefficients (≥0.3). Three factors were extracted for the pTESS-leg, in which all mental items were loaded on one separate factor with factor loadings exceeding 0.4. Active chemotherapy, less than one year from primary surgery, or tibial tumors were associated with significantly inferior pTESS/TESS scores in the lower extremity group.

**Conclusion:**

The Egyptian pTESS and TESS are valid and reliable self-reporting tools for assessing the functional outcome following surgeries for extremity bone sarcomas. The modified pTESS and TESS versions, which include additional mental domains, enabled the assessment of the overall health status of our population. Future studies should include a larger sample size and evaluate the ability of pTESS/TESS to track progress over time.

**Supplementary Information:**

The online version contains supplementary material available at 10.1186/s12955-023-02165-3.

## Introduction

### Background and significance

Osteosarcoma and Ewing sarcoma/Primitive neuroectodermal tumor (PNET) are the first and second most common types of malignant bone tumors that account for 6 percent of all childhood cancers [1–3]. Over two-thirds of bone sarcomas are primarily located in extremities, mostly affecting the lower limbs (57%), and less frequently occurring in the upper limbs (13%) [2, 3]. Surgery that involves a wide resection of malignant bone tumors is considered the mainstay of treatment, whereas chemotherapy is vital for improving 5-year event-free survival rates that can range from 60 to over 70% in localized bone cancer [4, 5]. In general, children with extremity bone sarcomas would undergo limb salvage surgery, such as allograft bone replacement or reconstruction with endoprosthesis; advanced cases are more prone to amputation, particularly with Osteosarcoma diagnosis in which radiotherapy is not an equivalent option for local control [6, 7]. Although surgery is crucial for cure, it contributes to a relatively higher rate of impaired physical function among survivors of childhood cancer [7, 8].

Few studies have reported patient-reported outcomes for children with bone sarcomas. They either used generic health-related quality of life (HRQOL) measures, such as the Pediatric Quality of Life Inventory (PedsQL) and the Pediatrics Outcomes Data Collection Instrument (PODCI), or disease-specific tools for evaluating functional outcomes after surgery, such as the Toronto Extremity Salvage Score (TESS) and the Patient-Reported Outcomes Measurement Information System (PROMIS) [8–13]. The generic measures could be missing important items for assessing the extent of physical disability, like pain, range of motion, and joint stability [14]. In contrast, a disease-specific tool would optimally evaluate the functional outcomes after local control in patients with bone sarcomas and assess the need for further treatment options, potential changes in lifestyle, or assistive devices [14]. Although TESS would be superior to generic HRQOL measures in assessing the physical domain of health status, it lacks a mental domain, which is essential in evaluating the overall HRQOL of these patients [15–18]. Previously reported TESS scores were highly correlated with the physical and social domains of the 36-Item Short Form Survey (SF-36), but not linked to its mental component score [17–19]. PROMIS is a single computerized adaptive testing (CAT) tool with several domains, including physical function and depression form, and it seems to be a proper choice that has been previously used following orthopedic surgeries [13, 20]. However, it might be inconvenient to offer a CAT tool in Egypt where computer illiteracy is expected to be prevalent among poorer residents; less than a quarter of Egyptian households in rural areas own a computer [21]. Adding a mental domain to the disease-specific TESS could be a simple alternative that would fit various cultures and circumstances, and its use would be convenient, especially in countries with lower levels of technological adoption.

Different models, with varying numbers of factors, were shown upon revealing the latent structure of TESS in previous studies; such results could be attributed to the existing socio-cultural differences and the close relation between the physical and the consequent social affection post-local control [18, 22–24]. This emphasizes the importance of further assessing the construct validity of TESS, especially after the addition of a mental domain, and evaluating its psychometric properties compared to the original TESS.

In addition, TESS was originally developed for an age group ranging from 12 to 60 years, and it includes items that seem irrelevant for children and adolescents [14, 25]. Accordingly, the pediatric Toronto Extremity Salvage Score (pTESS) was developed and validated to be used for North American patients aged from 8 to 17.9 years [25]. The pTESS needs to be evaluated and validated across various pediatric populations, similar to the cross-cultural adaptation and validation of TESS that have been done in several countries [15, 16, 18, 19, 22, 23, 26–28]. This pediatric version could also be modified to include a mental domain to reflect on the overall health status of children with bone sarcoma. Unlike PedsQL and other generic tools for children, the modified pTESS would act as a single tool assessing both the extent of physical disability and HRQOL for children with bone sarcoma in extremities. Thus, it is necessary to assess the reliability and validity of the modified pTESS version to implement and extend its use across different pediatric groups as a HRQOL measure specific for childhood bone cancer.

In a single center in Egypt, different surgical modalities are feasible for patients with extremity bone sarcomas, such as vascularized autograft, adult prosthesis, minimally invasive expandable prosthesis, and rotationplasty; due to the high cost and unavailability, non-invasive expandable prosthesis and allograft bone replacement are not used [29, 30]. The Musculoskeletal Tumor Society (MSTS) scoring system is the only routine measure for assessing functional outcomes in this population [29]. Although MSTS is disease-specific, it is not a patient-reported tool lacking the patients’ perceptions of their outcomes and increasing the risk of assessment bias [14, 17, 31]. The recently validated Egyptian version of PedsQL represents a great opportunity for evaluating the validity of other disease-specific and patient-derived tools like pTESS/TESS [32]. Accordingly, the cultural adaptation and validation of the original pTESS and TESS and their modified versions, which include mental domains, would provide a single self-reporting instrument for assessing the functional outcome and HRQOL in Egyptians with extremity bone cancer. The modified pTESS/TESS would be more informative than using the original versions of pTESS/TESS and more convenient than relying on the generic PedsQL survey. The cultural adaptation and validation of these modified forms can be replicated in different countries, replacing the use of the original forms if they proved to be reliable and valid for assessing HRQOL.

### Specific aims

The primary aims of this study were to perform cross-cultural adaptation and validation of pTESS and TESS and evaluate the modified forms, which involve mental domains, as potential HRQOL measures specialized for patients with extremity bone sarcomas. While the secondary aims included measuring HRQOL among respondents who participated at different time points from the date of primary surgery and assessing differences in scores based on various characteristics of the respondents, such as age, gender, histological diagnosis, chemotherapy status, and tumor location.

## Methods

### Study design and setting

This is a cross-sectional study in which data were collected using the modified pTESS and TESS, which contain mental domains, in addition to the PedsQL 4.0 generic core instrument. Since the Arabic PedsQL had been previously validated in Egypt, its scores were used to validate the culturally adapted pTESS and TESS [32].

### Target population and survey methods

**Fig. 1:**
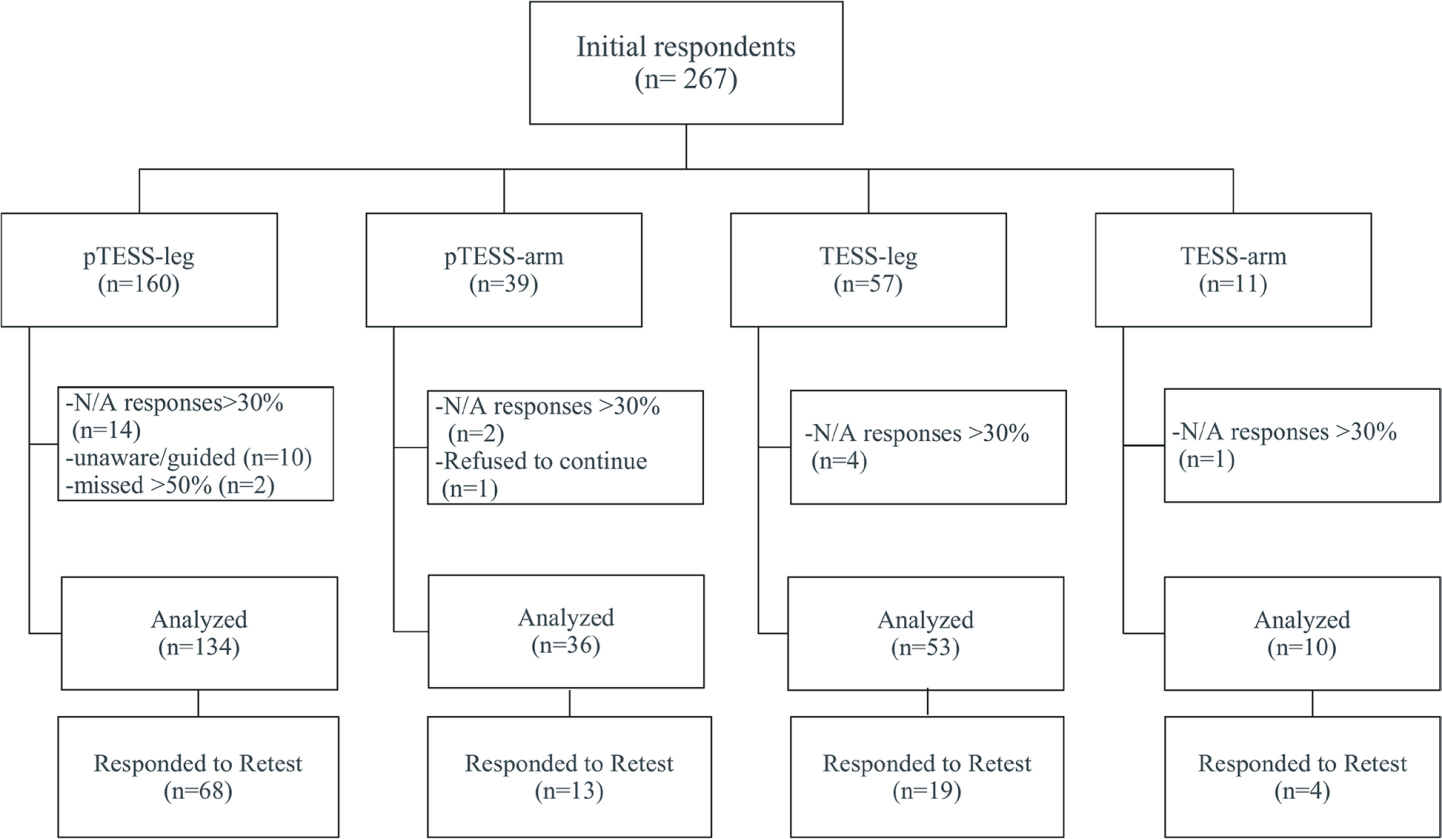
Flow diagram of the survey respondents

Patients were considered eligible for recruitment in the study if they had been diagnosed with Osteosarcoma or Ewing sarcoma/PNET of the upper or lower extremities in their childhood or adolescence, undergone primary surgery (+/-revision surgery) at least three months before the time of participation, visited the orthopedic clinic between January 2022 and June 2022, and were aged 8 years or over. Those who had progressive disease/relapse after surgery or were unable to fill out the questionnaires on their own were not eligible for the study. We also excluded participants who asked for significant guidance that was beyond clarifying a few words within the survey and those who had more than 25% of their pTESS/TESS responses as missing values or “not applicable” or missed over 50% of the PedsQL items (Fig. 1). The patient characteristics were readily available on REDCap software, a disease-specific registry (Table 1). For all the subgroups, we were able to reach most of the eligible patients during the study period, in which the largest group was expectedly pTESS-leg as bone sarcoma is more common in children and lower extremities (Fig. 1) [2]. Our sample also represented diverse characteristics in terms of age, gender, types of reconstruction, and time points from surgery (Table 1).

**Table 1:** Characteristics of respondents

Characteristic	Lower extremity		Upper extremity		Overall (%)
	**Pediatric (%)**	**Adult survivors (%)**	**Pediatric (%)**	**Adult survivors (%)**	
Eligible	134	53	36	10	233
Median age, years	14.3 (8–17.9)	20.6 (18–32)	13.3 (8–17.9)	20.8 (18–24)	15.6 (8–32)
Gender					
Male	70 (52)	27 (51)	14 (39))	5 (50)	116 (50)
Female	64 (48)	26 (49)	22 (61)	5 (50)	117 (50)
Diagnosis					
Osteosarcoma	91 (68)	41 (77)	9 (25)	4 (40)	145 (62)
Ewing sarcoma	43 (32)	12 (33)	27 (75)	6 (60)	88 (38)
Component					
Osseous	132 (99)	53 (100)	33 (92)	8 (80)	226 (97)
Extra-osseous	2 (1)	0	3 (8)	2 (20)	7 (3)
Tumor location					
Tibia	24 (18)	18 (34)	0	0	42 (18)
Femur	94 (70)	31 (58)	0	0	125 (54)
Fibula	13 (10)	4 (8)	0	0	17 (7)
Talus/Calcaneous	3 (2)	0	0	0	3 (1.5)
Humerus	0	0	19 (53)	8 (80)	27 (11.5)
Radius/ulna	0	0	3 (8)	0	3 (1.5)
Scapula	0	0	9 (25)	2 (20)	11 (5)
Shoulder/Clavicle	0	0	3 (8)	0	3 (1.5)
Metacarpal	0	0	2 (6)	0	2 (1)
Months from surgery	27.8 (3–156)	73.7 (3–169)	31 (3–135)	71.1 (25–112)	38 (3–169)
Type of LC					
Surgery	130 (97)	50 (94)	27 (75)	7 (70)	214 (92)
Surgery +RTH	4 (3)	3 (6)	0	1 (10)	8 (3)
ECI	0	0	9 (25)	2 (20)	11 (5)
Type of surgery					
Amputation	3 (2)	1 (2)	1 (3)	0	5 (2)
Rotationplasty	2 (1.5)	0	0	0	2 (1)
Limb salvage	129 (96.5)	52 (98)	26 (72)	8 (80)	215 (92)
Prosthesis	76 (59)	35 (67)	7 (27)	3 (37)	121 (56)
VFG	29 (22)	14 (27)	5 (19)	5 (63)	53 (25)
Non-VFG	0	0	2 (7)	0	2 (1)
Spacer/fixation	9 (7)	0	9 (35)	0	18 (8)
Fibulectomy/resection	15 (12)	3 (6)	3 (12)	0	21 (10)
Chemotherapy status					
On treatment/end <1 month	23 (17)	1 (2)	5 (14)	0	29 (12)
Finished treatment	111 (83)	52 (98)	31 (86)	10 (100)	204 (88)

For retests, we asked the respondents to answer the survey again by sending them a link to its electronic form via WhatsApp one week after their initial response. In delayed responses exceeding 2 weeks, participants were asked if their condition had changed in the test–retest interval.

### Instruments

As per the previously published guidelines, we performed a cross-cultural adaptation of pTESS/TESS forms [33, 34]. The initial two translations of pTESS and TESS were independently done by an informed translator (an orthopedic surgeon) and an uninformed translator (with a medical background). Then two independent back translations were done by a native professional translator and another bilingual individual with a medical background. The study members reviewed all the translated versions and agreed on further minor modifications before confirming the final Arabic forms of pTESS and TESS (Additional file 1). These modifications included simplifying some Arabic words to be readily understood by all age groups and educational levels. Moreover, some activities were adapted to fit our national context; “gardening”, in TESS, was broadened to “any agricultural activity, which may include basic farming activities” and “walking down or up a hill”, in pTESS/TESS-leg, was changed to “walking on steep roads” while “sexual activities” in TESS-leg was restricted to more conservative words, “intimate/marital relationships”.

TESS is a self-reporting questionnaire that was initially developed in English, then translated into other languages, cross-culturally adapted, and validated as a disease-specific tool in several studies [15, 16, 18, 19, 22, 23, 26–28]. The original TESS validation included a heterogeneous population who had a primary tumor in extremities, and the open-ended questions were used to decide on including additional relevant items [14]. In a subsequent representative sample that was evaluated longitudinally, the test–retest reliability, internal consistency, construct validity, predictive validity, and group validity measures of the original TESS were all shown to be satisfactory [14]. TESS includes two versions, one for the upper extremities (29 questions) and the other for the lower extremities (30 questions). Each question assesses the difficulty of performing a certain task related to dressing, work, or other usual physical and social activities. Because of our young adult population, we modified the questions, originally asking about work performance, to ask about either studies or work, whichever applies to the participant. The answers are basically ordered on a 5-point scale that originally starts with “Impossible to do”. However, we changed the order and wording of responses to be like pTESS where options start with “not hard at all” and end with “too hard I can’t do this”. All items also included a ‘not applicable’ option (N/A) that should be discarded when calculating the final standardized score, which ranges from 0 to 100; higher scores indicate better outcomes [14, 25].

The pTESS is a recent format that has been developed for assessing the functional outcome of the pediatric population. Its initial draft was evaluated using cognitive debriefing done by children with bone tumors. Unlike the original adult version, pTESS was not evaluated for its ability to detect progress in function over time; however, pTESS was shown to be reliable and valid using similar measures to those reported by the original TESS study [14, 25]. The total number of questions is 27 for the upper extremity version and 30 for the lower extremity version. The final scores of respondents were calculated exactly as per TESS [25].

Both modified versions of pTESS and TESS included the same additional mental domain, which involved six questions that were adopted from the pediatric anger, fatigue, cognitive, and depression domains of the Neuro-QOL system as well as the mental component of SF-36 [35, 36]. The possible responses to each of the mental items represent a 6-point ordinal scale. To follow the same standardized scoring used for the original pTESS and TESS, we rescaled these items to a 5-point range without changing the number of possible responses [37]. Rescaled values were only used upon calculating the total standardized score, while the original scale was used in descriptive analyses to avoid confusion during data interpretation. It takes from 10 to 15 min to complete the modified pTESS/TESS.

The PedsQL 4.0 generic core survey was used to measure the HRQOL of all participants. It takes about 4 to 5 min to complete the four covered domains: physical, emotional, social, and school/work functioning. The adult forms of PedsQL were used for those who were ≥ 18 years old at the time of the survey. Any missing responses in PedsQL were considered invalid and excluded from the final scores which range from 0 to 100, with higher scores indicating superior HRQOL [32, 38].

### Validation and statistical analysis

Patients were asked about the relevance of pTESS/TESS in assessing their HRQOL. The two open-ended questions found in the original pTESS were also included in our pTESS and TESS forms to check if there were other relevant items not covered by the questionnaire. The total standardized scores were calculated for the pTESS and TESS forms. Responses to the mental items were excluded while computing the final scores of the original pTESS/TESS versions. We tested the difference between scores of the original pTESS/TESS and modified pTESS/TESS using paired t-test. The internal consistency of each version was assessed by Cronbach’s alpha, in which pairwise deletion of N/A and missing responses was done instead of list-wise deletion to prevent dropping several valid cases from the analysis. We also checked the occurrence of floor or ceiling effects by identifying whether more than 15% of participants obtained the highest or lowest possible score, respectively [39]. The test–retest reliability was evaluated using the intra-class coefficient (ICC) values based on the criteria suggested by Koo & Li [40]. For construct validity, an exploratory factor analysis (EFA) was intended to examine the grouping of items after adding the mental domain. The varimax rotation method was chosen, and the rotated factor loadings were considered acceptable if they exceeded 0.4. The extracted number of factors was based on Kaiser’s criteria and observing the “elbow” point in a scree plot [41, 42]. However, we were able to conduct EFA for the pTESS-leg only, as other versions involved insufficient numbers of participants. Thus, we have only checked if the average inter-item correlation coefficient for the remaining groups fell between 0.3 and 0.7 [43, 44]. Confirmatory Factor Analysis (CFA) was done to confirm the EFA-suggested model in which an acceptable fit model is considered if the comparative fit index (CFI) is ≥0.95, the root mean square error of approximation (RMSEA) is ≤0.08 with an upper CI bound of 0.10, and the standardized root mean squared residual (SRMR) is ≤0.08 [45]. In factor analysis, pairwise deletion of missing values was done as no pattern was observed in the missing data, and they were, together with N/A responses, less than 5% in all versions [46–48]. For convergent validity, we assessed the correlation between the pTESS/TESS scores and the PedsQL scores [43]. Categories for strength of correlation were either weak (0.1–0.3), moderate (0.31–0.69), strong (0.7–0.9), or very strong (0.91–1) [49, 50]. Moreover, differences in total scores based on patient characteristics were examined using the Mann–Whitney U test. For variables involving more than two groups, the Kruskal–Wallis H test was used instead. The number of participants who underwent amputation or rotationplasty was very small (*n*=6); therefore, they were excluded when comparing differences in scores based on other factors to avoid affecting the results of the limb salvage surgery group, which represents the largest group of patients. Those who had temporary spacers in their lower extremities were also excluded from any analyses other than the survey validation since future improvement is expected after reconstruction. Alternatively, spacers in the upper extremities would offer a shoulder function similar to the definite method of reconstruction, making their corresponding cases eligible for inclusion during the assessment of secondary aims. All the statistical tests were carried out using SPSS software (version 20) and R statistical environment (version 3.4.4).

### Ethical considerations

This study was approved by the institutional review boards of CCHE-57357 and the American University in Cairo. Written consent forms were obtained from participants and/or their legally authorized representatives, depending on the respondents’ age.

## Results

### Respondents’ characteristics

In a total sample of 267 participants, only 233 were included in the analysis of which 187 answered the pTESS/TESS for the lower extremity, while 46 completed the upper extremity forms. In both groups of upper and lower extremities, the median age of adults was over 20 years. Pediatrics of the upper extremity had a slightly lower median age of 13.3 years compared to 14.3 years in the lower extremity group. The humerus and femur bones were the most common sites in the upper and lower limbs, respectively. Most of the participants (88%) had finished chemotherapy at the time of the survey (Table 1).

### Total scores and item responses in upper and lower extremities

For the 187 participants who answered the lower extremity forms, either pTESS or TESS, and the other 46 who responded to the upper extremity survey, the median (IQR) scores of the modified versions were 69.2 (20.5) and 73.1 (20.2), respectively. While the median scores of the original versions were 72.5 (21.9) for the lower extremity and 76.0 (23.4) for the upper extremity. The original versions represented significantly higher scores compared to the modified pTESS/TESS versions (*p*-value <0.001).

Out of the 187 respondents with lower extremity sarcomas, 134 patients completed the pTESS and 53 answered TESS (Table 1). The average score among the original items of pTESS was 3.7. As shown in Table 2, the lowest median score per item was equal to 1, and it was found with “running” (question 28). The average score across all additional mental questions was 4. Regarding the TESS-leg form, the average item score was 3.95 for the original items and 3.2 for the mental items. The lowest median score per item was 3, but mode scores as low as 1 were noted for “kneeling” (question 13) and “getting up from kneeling” (question 23). Across the mental questions, “Do you easily get in a bad mood?” (question 35) had the lowest median score, which was equal to 3.

**Table 2:** Median and mode scores of pTESS and TESS

	pTESS leg		TESS leg		pTESS arm		TESS arm	
	Median	Mode	Median	Mode	Median	Mode	Median	Mode
Question 1	5	5	5	5	5	5	5	5
Question 2	5	5	4	5	5	5	5	5
Question 3	5	5	5	5	5	5	5	5
Question 4	5	5	5	5	5	5	5	5
Question 5	5	5	4	5	5	5	4	3
Question 6	3	1	N/A^a^	N/A^a^	4.5	5	5	5
Question 7	5	5	5	5	5	5	5	5
Question 8	3	3	5	5	5	5	5	5
Question 9	5	5	3	3	5	5	5	5
Question 10	5	5	5	5	5	5	3.5	3
Question 11	5	5	5	5	4	3	5	5
Question 12	3	5	5	5	4	5	4.5	3
Question 13	5	5	3	1	4	5	5	5
Question 14	4	3	4	5	5	5	4	4
Question 15	4	5	4	4	2.5	2	5	5
Question 16	3	1	4	5	4	5	3	3
Question 17	5	5	N/A^a^	N/A^a^	3.5	5	5	5
Question 18	4	5	5	5	4	5	4.5	5
Question 19	4	3	4	4	2	1	3.5	3
Question 20	5	5	5	5	4	5	2.5	5
Question 21	3	1	3	3	5	5	5	5
Question 22	5	5	5	5	5	5	5	5
Question 23	5	N/A	3	1	5	5	5	5
Question 24	5	5	4	5	5	5	5	5
Question 25	4	5	N/A^a^	N/A^a^	5	5	4.5	5
Question 26	5	5	4	3	5	5	4.5	5
Question 27	4	1	4	5	4.5	N/A	5	5
Question 28	1	1	5	5	3	2	5	5
Question 29	3	1	5	5	4	6	4.5	5
Question 30	2	1	5	N/A	4	6	3.5	4
Question 31	4	2	4	1	4	1	5	5
Question 32	5	6	4	4	3.5	6	4.5	6
Question 33	5	6	4	5	3	1	4.5	5
Question 34	5	6	5	6			5	5
Question 35	4	6	3	3			5	5
Question 36	4	6	4	4				
Total score	68.2		71.9		72.7		80.3	
Original score^b^	69.3		77.0		76.0		81.0	

^a^Excluded from the total score (as internal consistency improved upon this item removal)

^b^Standardized score of original versions

As for the upper extremity group, 36 were included in the analysis of the pTESS-arm. The average score among the original items and the mental items was 3.7 and 3.4, respectively. The lowest median scores were 2 and 2.5, which were obtained with only two questions; “carrying heavy things” (question 15) and “lifting a box to a high shelf’ (question 19). Only 10 respondents were analyzed in the TESS-arm group, and they had an average item score of 4.2 in each of the original and the mental domain. The lowest median score per item was 2.5, and it was only found with “lifting a box to a high shelf” (question 20) (Table 2).

### Validation

In the open-ended questions of each pTESS/TESS form, over 80% of participants denied the presence of more relevant tasks other than those mentioned in the survey; 7 (13%) participants in TESS-leg added that running/prolonged walking had become difficult, and 9 (6.7%) in pTESS-leg stated difficulties in performing Islamic prayer movements. In all forms combined, 6 (2.6%) respondents added comments related to swimming, and 25 (10%) revealed that the appearance of surgical scars is annoying to them.

The Cronbach's alpha exceeded 0.9 in all the tested versions (Table 3). In TESS-leg, omitting questions 6 (gardening), 17 (driving), and 25 (participating in sexual activities) increased the Cronbach’s alpha from 0.78 to 0.92. These questions were chosen by more than half of the respondents as “N/A.”. The remaining versions did not show an improvement in internal consistency upon removing any items. All versions also revealed good test–retest reliability with ICC values > 0.8 (Table 3). Moderate to strong correlation coefficients that range from 0.55 to 0.86 resulted from testing PedsQL scores against the scores of pTESS and TESS. As shown in Table 3, the inclusion of mental scores enhanced the correlation between PedsQL scores and lower extremity scores in both pediatric and adult forms. Total scores have not shown a floor or ceiling effect in any of the tested versions.

**Table 3:** Validation of pTESS and TESS

		**Lower extremity**				**Upper extremity**				
	α ^a^	IIC^b^	ICC^d^	r-PedsQL^e^	*p*-value	α ^a^	IIC^b^	ICC^d^	r-PedsQL^e^	*p*-value
PTESS	0.94	0.33		0.65	<0.001	0.93	0.35		0.638^f^	<0.001
Modified pTESS	0.94	0.4^c^	0.824	0.75	<0.001	0.93	0.51^c^	0.834	0.64^f^	<0.001
TESS	0.92	0.3		0.551	<0.001	0.94	0.37		0.836	0.003
Modified TESS	0.93	0.54^c^	0.822	0.751	<0.001	0.94	0.49^c^	0.828	0.858	0.001

^a^Cronbach’s alpha- raw alpha was calculated for pTESS and TESS,—standardized alpha was calculated for Modified version of pTESS/TESS

^b^The Average Inter-Item Correlation coefficient

^c^ The IIC of mental domains only

^d^ Intraclass coefficient

^e^Correlation with PedsQL (Pearson correlation coefficient except ^f^)

^f^ Spearman correlation coefficient

**Fig. 2:**
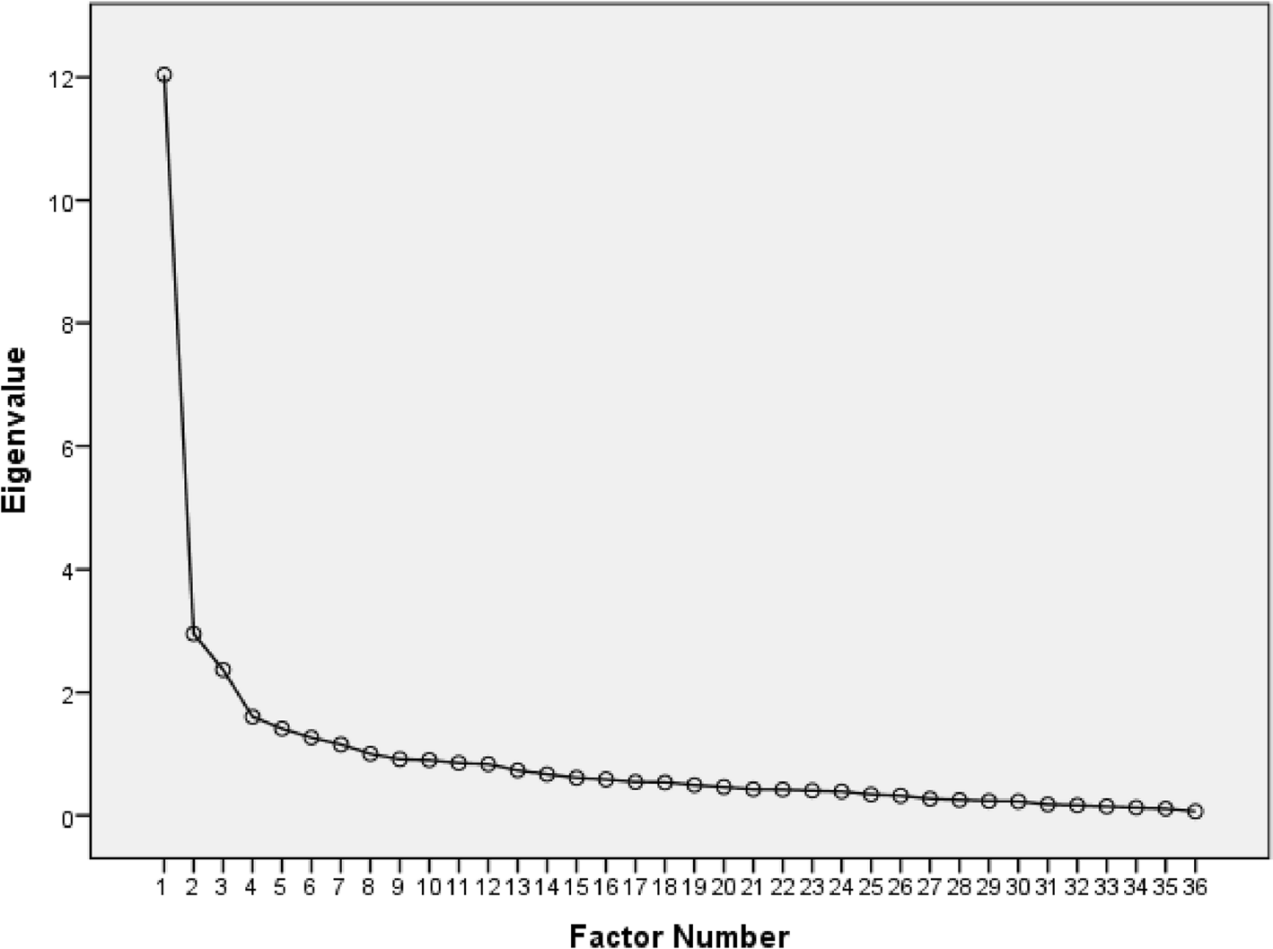
Scree plot for pTESS-leg

**Fig. 3:**
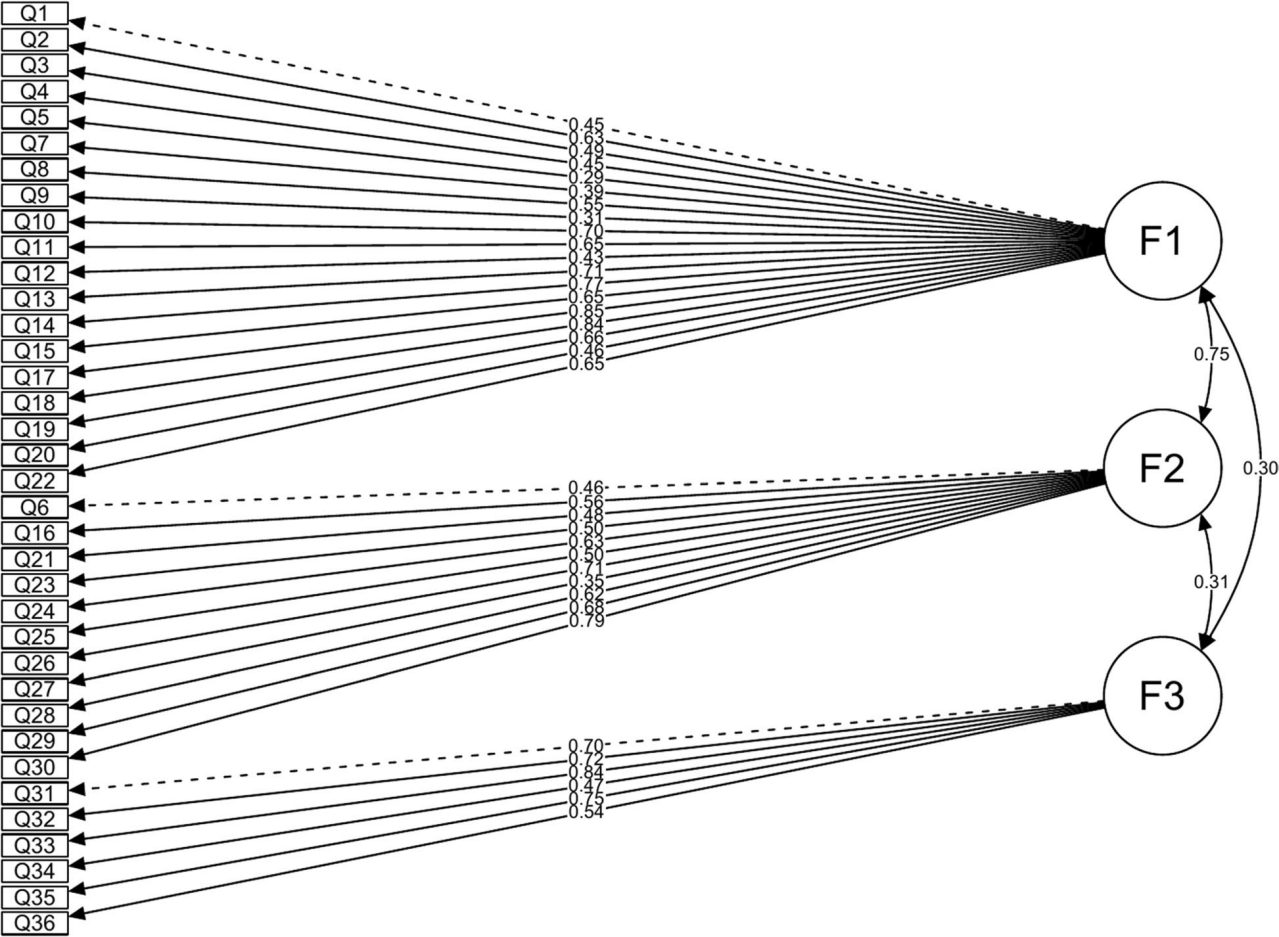
Path diagram of the CFA model for pTESS-leg

In addition, the average inter-item correlation coefficients for the original pTESS and TESS as well as their mental domains were all within the desirable range (0.3–0.7) (Table 3). Upon performing EFA for pTESS-leg, 3 factors were extracted based on the clear elbow shown in the scree plot (Fig. 2). All the mental items were loaded on a separate factor with factor loadings that ranged from 0.43 to 0.77. Questions numbers 16, 21, and 23 to 30 loaded on factor 2; these questions are generally related to social interaction or relatively harder physical activities (Additional file 2). Walking upstairs (question 14) or up/down a hill (question 19) cross-loaded on factors 1 and 2. The items that demonstrated weak factor loadings, below 0.4, were bending down on knees (question 12) and standing straight (question 20) (Additional file 2). The 3-factor model was confirmed by CFA, in which CFI was 0.954, RMSEA was 0.072 with the upper 90% CI bound equal to 0.081, and SRMR was 0.099. As expected, a stronger correlation is shown between the first 2 factors compared to their correlation with factor 3, which represents the mental domain (Fig. 3).

### Children and adolescents versus adults

The median scores of the modified pTESS and TESS of the lower extremities were 68.2 and 71.9, while their equivalent original version scores were 69.3 and 77.0. The difference between pediatric and adult groups was statistically significant in the original version scores only (*p*-value = 0.038). In the upper extremities, the modified pTESS and TESS median scores were 72.7 and 80.3, respectively, while their corresponding original scores were 76.0 and 81.0. No statistically significant differences were revealed upon comparing the scores of pTESS and TESS in the upper extremities.

### Scores based on respondents’ characteristics

In the lower extremities, the scores of both the modified and original versions that were obtained prior to reaching one year from surgery were significantly lower than those obtained beyond one year from surgery (*p*-value = 0.001, <0.001) (Table 4). No significant improvement was shown 2 to 8 years after surgery. Conversely, the duration since primary surgery did not affect any of the upper extremity scores (Table 4). Other factors that showed a statistical difference in the lower extremity group were the chemotherapy status and tumor site (*p*-value = 0.047, 0.002). Those who had finished chemotherapy or had their tumors located in the fibula and femur but not the tibia showed favorable outcomes in terms of both modified and original pTESS/TESS scores (Table 4).

**Table 4:** Total scores in the limb salvage group

	**n**	**LE- Modified**^**a**^		**LE- Original**^**b**^		**n**	**UE- Modified**^**a**^		**UE- original**^**b**^	
		Median (IQR)	*P*-value	Median (IQR)	*P*-value		Median (IQR)	*P*-value	Median (IQR)	*P*-value
Overall^c^	172	69.2 (20.5)		73.0 (21.7)		45	73.1 (20.0)		76.1 (22.3)	
Age group			0.124		0.04*			0.239		0.492
Pediatric	120	68.5 (20.6)		70.7 (23.9)		10	80.3 (31.7)		81.0 (30.9)	
Adult	52	72.6 (19.0)		77.0 (19.4)		35	73.0 (17.3)		76.1 (19.1)	
Gender			0.809		0.38			0.175		0.383
Male	86	69.3 (22.3)		71.8 (24.5)		19	75.0 (23.8)		81.5 (23.1)	
Female	86	69.1 (16.3)		73.1 (24.5)		26	72.7 (22.8)		75.9 (29.3)	
Diagnosis			0.077		0.204			0.99		1
Osteosarcoma	120	67.3 (21.2)		71.2 (24.3)		32	73.8 (19.7)		76.5 (20.4)	
Ewing sarcoma	52	72.9 (15.9)		74.6 (19.3)		13	72.3 (25.8)		76.1 (28.2)	
Tumor location^d^			0.002*		0.007*			0.427		0.293
Tibia	41	61.5 (21.7)		65.4 (26.1)						
Femur	112	70.9 (18.1)		73.2 (20.6)						
Fibula	17	73.5 (14.7)		76.0 (13.9)						
Humerus						27	70.2 (16.2)		72.6 (26.6)	
Scapula						11	74.5 (19.1)		77.1 (22.7)	
≥ 1 year from surgery			0.001*		< 0.001*			0.561		0.819
No	32	63.1 (27.9)		63.0 (28.4)		10	75.0 (17.3)		72.8 (18.4)	
Yes	140	70.8 (19.4)		74.1 (19.9)		35	73.0 (22.0)		76.9 (29.2)	
Limb salvage^d^			0.308		0.17			0.506		0.369
Prosthesis	111	69.1 (19.4)		72.9 (19.8)		10	72.2 (12.4)		74.4 (19.3)	
VFG	43	68.1 (24.9)		72.2 (26.4)		10	69.0 (35.7)		74.0 (36.5)	
Fibulectomy	16	73.2 (12.5)		75.5 (12.6)						
ECI						11	74.5 (19.1)		77.1 (22.7)	
Chemotherapy			0.047*		0.023*			0.93		0.428
On therapy^e^	20	64.9 (24.7)		63.6 (26.8)			68.4 (18.0)		69.2 (19.7)	
Ended therapy	152	70.2 (21.5)		73.2 (22.6)			73.8 (20.9)		77.0 (23.0)	

^a^Modified versions of pTESS/TESS

^b^Original versions of pTESS/TESS

^c^Temporary spacers of lower extremity were excluded

^d^Talus and calcaneous (*n*=2) were excluded from this comparison

^e^Those who ended treatment in less than one month were included in “on therapy” group

### PedsQL measures

The mean±SD (median) of PedsQL scores for pediatric lower extremity and upper extremity were 57.3±18.1 (58.3) and 59.8±22.5 (59.2), respectively. While the mean±SD (median) adult lower and upper scores of PedsQL were 66.1±23.1 (67.9) and 77.5 ± 13.9 (80.1). Although TESS-leg (the original version) relatively showed a weaker correlation with PedsQL, in which the correlation coefficient was only equal to 0.55 (Table 3), a considerably enhanced correlation resulted upon evaluating TESS-leg to the physical domain of PedsQL only (*r*=0.687, *p*-value <0.001).

## Discussion

### Overview

There is a lack of validated tools for measuring the functional outcome and HRQOL following surgeries for childhood bone sarcomas. We performed cross-cultural adaptation and validation of the pTESS, to be used for Egyptian pediatric patients, and TESS, to be used for adult survivors of childhood bone cancer. In addition, this is the first study to include a mental domain in pTESS/TESS and assess the reliability and validity of these modified versions for their potential use as HRQOL measures specific for extremity bone sarcoma. All versions showed no floor or ceiling effects, excellent internal consistency, and high test–retest reliability. The moderate to strong correlations with PedsQL scores confirmed the convergent validity.

### The modified pTESS/TESS and HRQOL

Modifying pTESS and TESS to additionally assess the mental status and reflect on HRQOL has shown promising results that encourage further validation in future studies and replication of these modified versions in other settings. Compared to the original pTESS/TESS, stronger correlations were shown between the modified pTESS/TESS and PedsQL, which represents the generic HRQOL tool. This result indicates that the added mental domain enhanced the ability of pTESS/TESS to evaluate HRQOL, not only the functional outcome, which confirms the convergent validity of the modified pTESS/TESS. Such a need for overall health status assessment was previously recognized by Ogura et al. and Xu et al. [18, 22]. However, the current scores of mental status should be interpreted with caution, noting that the CFA model has shown weak correlation between the mental domain and the other two domains related to physical and social activities. Our mental scores seem to provide an overview of the general mental condition that is not confined to mental issues specifically related to the affected physical function. Pre-existing and/or coexisting conditions related to the disease could have had a great impact on the resulting mental scores. Moreover, the way patients perceives their physical affection could have a significant mental impact; adapted individuals would maintain a better mental state even with a major physical dysfunction, while less adapted ones with a favorable functional outcome can still feel greatly disabled [51]. Therefore, these varying perceptions could also explain the weak correlation between the mental and physical/social domains. Although the causes of our mental scores are currently not well defined, the significantly higher scores of the original pTESS/TESS versions, compared to the total scores of their modified versions, suggest a considerable psychological affection that is worth further assessment when evaluating the HRQOL for patients undergoing surgeries for bone sarcomas. HRQOL measures, including mental status, could aid the orthopedic surgeons in assessment and decision making; for instance, deciding on patient eligibility for different treatment options, choosing how to deal with patients and their possible need for special assistance, or interpreting various responses to treatment with the role of mental health in affecting such responses [52]. To better interpret the mental scores in the future, it could be useful to include more specific items that indicate whether the declared mental feelings are general or directly related to physical disability.

### pTESS

Regarding pTESS, the median scores of the original versions in this study were lower than the mean scores reported by Piscione et al.: 76 versus 81 in the arm and 69 versus 77 in the leg [25]. However, the internal consistency and reliability were comparable in both studies [25]. Such validity measures were also kept favorable upon further assessment of our modified versions. Different methods are considered valid for assessing the construct validity; in the original pTESS study, the authors relied on hypothesis testing to evaluate the construct validity, while in our study, we aimed to explore the structure of the modified pTESS-leg and observe the factor loadings for the originally existing items and the additional mental items [25, 43]. Upon conducting EFA, “standing straight” seemed to be the most irrelevant item; it represented the lowest factor loadings and was highly cross-loaded across 2 of the 3 demonstrated factors. Having a convenient solution for this complication, such as using a shoe lift to correct limb length discrepancies, could explain the irrelevance of this item to other activities that are less likely to be simply enhanced by a minor intervention or nonsurgical treatment. “Bending down on knees” is another item with low factor loadings, and it might require clarifying the degree of kneeling and revisiting the translated Arabic words in future assessments. Conversely, the six mental items were perfectly loaded within the same factor, which verified the validity of this extra domain. Even though the pTESS-leg model is considered to have an acceptable fit, re-performing EFA and/or CFA on a larger sample is required to confirm the current results, especially given that the number of items is relatively high and the SRMR exceeded the expected value [53]. As for the pTESS-arm, its current validity measures are promising since their values were quite acceptable despite the few responses. Such results were also analogous to previously reported pTESS measures [25]. However, the power of the performed analyses was probably affected by the small sample size, and a higher number of respondents in this group is needed to confirm the current results and permit conducting factor analysis for the modified version of pTESS-arm.

### TESS

The consistency and reliability of all our TESS versions (the original and modified ones in arm and leg) showed similar results to those reported in the initial TESS study [14]. In contrast, our TESS versions have shown stronger correlations with PedsQL compared to the previously reported correlation with MSTS [14]. It is important to note that the internal consistency was considerably improved upon removing questions 6, 17,and 25. The irrelevance of these questions might be explained by the existing cultural differences [14, 23, 34]. Gardening (question 6) seems to be an uncommon activity in Egypt, and even though the word “gardening” was translated into an Arabic word that could also mean farming, several participants answered that they don’t do any of these agricultural activities. Moreover, most of the respondents were young adults who are less likely to engage in sexual activities (question 25) or learn to drive at their current age (question 17). Therefore, these three questions might be discarded in future assessments. Unlike our study approach, the initial TESS study was able to confirm the predictive validity of TESS by evaluating the participants at multiple time points, which proved the ability of TESS to detect changes over time and in response to treatment interventions. Regarding the total scores, our original TESS-leg version revealed an Egyptian median score that was slightly higher than those reported in Italy and Greece [16, 23], and comparable to Vienna [19], while being inferior to several other scores [15, 18, 26–28]. The heterogeneity in study designs, age groups, diagnoses, tumor sites, and treatment modalities makes it harder to compare these findings. Stish et al. found that adults with pediatric Ewing sarcoma had higher scores than those who were adults at the time of diagnosis [54]. For better assessment, this should be further evaluated with the inclusion of Osteosarcoma diagnosis and a higher number of participants. Moreover, Stish et al. have not mentioned the mean age of respondents below 18 years old [54]. Stating the mean age would have provided a more valuable interpretation, as surgeries done in preadolescence are expected to be more challenging than those done in adolescence and adulthood [55]. Accounting for age could have explained our inferior scores reported by survivors of preadolescent surgeries who have probably faced multiple revision surgeries before filling out the survey. Hence, investigating the impact of the number of revision surgeries on HRQOL would also be useful in future studies. Moving to TESS-arm, fewer studies are available for this group. Our TESS-arm median score was again similar to that of Vienna [19], but lower than other scores [15, 16, 22, 28]. This rare subgroup requires further research that would pool data from multiple centers to reach a sufficient sample size. Although TESS-arm represented the smallest group in our study, having only ten participants, the resulting validity measures were quite favorable, which encourages the assessment of the current TESS-arm version in future studies.

### Pediatric and adult participants

Since the gap between pediatrics and adults for the lower extremity group was reduced upon including the mental domain scores, long-term psychological effects that could last beyond the improvement of physical function might be considered. Another possibility is that adults are generally more aware and well-informed of their health conditions which could affect their mental health to a greater extent [56]. The absence of any differences in the upper extremity group is possibly due to the small sample size or the chance that fewer severe complications would result from upper limb surgeries and affect mental health [55].

### Responses and different characteristics

Exceeding one-year post-surgery in the lower extremity group had been associated with better outcomes, while no significant difference was found at later time points after surgery. This result was consistent with previous findings that showed significant enhancement after one year of surgery but minor improvements in the functional outcome and HRQOL 2 to 7 years later [9, 12, 57]. Chemotherapy was linked to worse outcomes in the lower extremities; this was the case in previous measures for both pediatric and adult groups [11, 25]. However, the fact that those who were still receiving chemotherapy had been at earlier time points from surgery could be a significant confounder. Thus, a higher number of diverse respondents is required to enable multivariable analysis in future studies. Regarding the tumor site, the inferior scores with the tibia bone can be attributed to the higher incidence of various complications in proximal tibial resections, like infection and inferior knee range of motion due to reattachment of the extensor mechanism during primary surgery [58, 59]. Overall, a larger sample size and a prospective analysis would provide a better interpretation of the impact of different characteristics on HRQOL in childhood bone cancer, especially in the upper extremities.

The few participants with amputation surgeries prevented us from comparing their outcomes to limb salvage surgeries. Piscione et al. have not found differences in pTESS scores between both groups [25]. Nevertheless, other previous studies have shown conflicting results regarding HRQOL following limb salvage surgeries versus amputation [27, 60–62]. This contradiction emphasizes the importance of comparing different types of surgeries within our studied population to determine its own preferences.

In addition, our reported PedsQL scores have been shown to be lower than those of healthy children in Egypt but similar to the mean scores of Egyptian children with chronic conditions [32]. This deviance from the healthy population emphasizes the need for the evolving advanced surgical techniques and individualized tools that are being introduced in orthopedic oncology for the aim of enhanced outcomes [63, 64].

### Limitations

Besides the small sample size of the upper extremity groups and the adult leg group, there were other limitations in this study. Owing to its cross-sectional nature, the ability of pTESS and TESS to detect changes in outcomes over time still needs to be investigated. Moreover, a relatively higher proportion of excluded respondents belong to the pediatric group, which suggests that a self-reporting tool can be challenging and that its scores might not be fully representative of the younger population. Not to mention that the long-term effects of chemotherapy, such as cardiotoxicity, and the Coronavirus Disease 2019 (COVID-19) could have interfered with our findings, especially in moderate-to-vigorous physical activities or outdoor-related items. Finally, socio-economic status was not evaluated in this study, even though it could have affected the outcome measures.

## Conclusion and future work

Our culturally adapted versions of pTESS and TESS are considered valid and reliable self-reporting tools for Egyptians with childhood bone sarcomas in extremities. This study has been the first to modify pTESS/TESS by including a mental domain and validate such forms as a HRQOL measure. This allows for a single disease-specific tool that is able to accurately assess the functional outcome and reflect on HRQOL at the same time. It is recommended to further study whether routinely obtaining patient-reported outcomes would enable healthcare providers to monitor the patient’s physical and mental progress over time. A larger sample size is required to verify the current findings and enable the stratification of HRQOL measures by local control modality to aid in clinical decision-making. We believe that the modified pTESS/TESS versions have provided an added benefit for evaluating the overall health status of patients and survivors of childhood bone sarcoma, which is quite important in the initial assessment, treatment planning, outcome evaluation, and possible consideration of innovative surgical techniques. Our promising results would encourage further validating the modified pTESS/TESS and extending their use to other countries as self-reporting tools for HRQOL.

## Supplementary Information

**Additional file 1.** The Egyptian version of pTESS/TESS.

**Additional file 2.** Rotated factor matrix and plot.

## Data Availability

The datasets used and/or analysed during the current study are available from the corresponding author on reasonable request.

## Abbreviations

CAT: Computerized adaptive testing
CFI: Comparative fit index
COVID-19: Coronavirus disease 2019
EFA: Exploratory factor analysis
CFA: Confirmatory factor analysis
HRQOL: Health related quality of life
ICC: Intraclass coefficient
IQR: Interquartile range
MSTS: Musculoskeletal tumor society
N/A: Not applicable
PedsQL: Pediatric quality of life inventory
PNET: Primitive neuroectodermal tumor
PODCI: Pediatrics outcomes data collection instrument
PROMIS: Patient-reported outcome measurement information system
pTESS: Pediatric toronto extremity salvage score
pTESS-arm: Pediatric Toronto extremity salvage score of upper extremity
pTESS-leg: Pediatric Toronto extremity salvage score of lower extremity
RMSEA: Root mean square error of approximation
SD: Standard deviation
SF-36: 36-Item short form survey
SRMR: Standardized root mean squared residual
TESS: Toronto extremity salvage score
TESS-arm: Toronto extremity salvage score of the upper extremity
TESS-leg: Toronto extremity salvage score of the lower extremity
