# Supplementary material for: Cross-cultural adaptation and validation of a self-reporting tool to assess health-related quality of life for Egyptians with extremity bone sarcomas in childhood or adolescence
Source: Health Qual Life Outcomes. 2023 Jul 29;21:81. doi: 10.1186/s12955-023-02165-3 (PMC10386605; doi:10.1186/s12955-023-02165-3)
Supplement: Supplementary file 1 — Additional file 1. The Egyptian version of pTESS/TESS. [file 12955_2023_2165_MOESM1_ESM.pdf]

Additional file 1

The Egyptian version of pTESS/TESS

استبيان ما بعد جراحات انقاذ الأطراف
استبيان تورونتو المعدل للأطفال
استبيان للساق
Pediatric Toronto Extremity Salvage Score
(pTESS-Leg)

في الاسئلة اللي جاية هنسالك علي بعض الاشياء بتعملها في الحياة و عايزين نعرف اذا كان سهل عليك انك تعملها الاسبوع اللي فات. ممكن تكون في حاجات مقدرتش تعملها لكن مفيش مشكلة. عايزينك تختار الاجابة اللي تحس انها صح بالنسبة لك.

لو سمحت لا تطلب من والديك مساعدتك في الإجابات. لو محتاج مساعدة في قراءة كلمة ، ممكن تطلب منهم قراءتها لك ، ولكن لازم تجاوب بنفسك.

مثال:

ركوب العجلة :

**1** مش صعب ابدا	**2** صعوبة بسيطة	**3** صعوبة متوسطة	**4** صعب جدا	**5** صعب جدا و لا استطيع فعل هذا ابدا	**6** لا أفعل هذا

لو في حاجة كنت متعود تعملها لكن بسبب رجلك مش قادر تعملها دلوقتي اختار الاجابة رقم "5 "صعب جدا. لا أستطيع أن أفعل هذا" لو مش متعود تعمل النشاط ده ، اختار الإجابة "6".

الإصدار: ديسمبر 2021

عايزين نعرف اذا كان من السهل عليك القيام بالأنشطة التالية الأسبوع الي فات:

1) لبس البنطالون :

**1** مش صعب ابدا	**2** صعوبة بسيطة	**3** صعوبة متوسطة	**4** صعب جدا	**5** صعب جدا و لا استطيع فعل هذا ابدا	**6** لا أفعل هذا

2) لبس الحذاء:

**1** مش صعب ابدا	**2** صعوبة بسيطة	**3** صعوبة متوسطة	**4** صعب جدا	**5** صعب جدا و لا استطيع فعل هذا ابدا	**6** لا أفعل هذا

3) لبس الشراب:

**1** مش صعب ابدا	**2** صعوبة بسيطة	**3** صعوبة متوسطة	**4** صعب جدا	**5** صعب جدا و لا استطيع فعل هذا ابدا	**6** لا أفعل هذا

4) الاستحمام من غير مساعدة احد:

**1** مش صعب ابدا	**2** صعوبة بسيطة	**3** صعوبة متوسطة	**4** صعب جدا	**5** صعب جدا و لا استطيع فعل هذا ابدا	**6** لا أفعل هذا

5) تنظيف غرفتك:

**1** مش صعب ابدا	**2** صعوبة بسيطة	**3** صعوبة متوسطة	**4** صعب جدا	**5** صعب جدا و لا استطيع فعل هذا ابدا	**6** لا أفعل هذا

6) القفز او النط علي رجل واحدة:

**1** مش صعب ابدا	**2** صعوبة بسيطة	**3** صعوبة متوسطة	**4** صعب جدا	**5** صعب جدا و لا استطيع فعل هذا ابدا	**6** لا أفعل هذا

7) المساعدة في التسوق

**1** مش صعب ابدا	**2** صعوبة بسيطة	**3** صعوبة متوسطة	**4** صعب جدا	**5** صعب جدا و لا استطيع فعل هذا ابدا	**6** لا أفعل هذا

الإصدار: ديسمبر 2021

8) تحريك الحاجات الثقيلة:

**1** مش صعب ابدا	**2** صعوبة بسيطة	**3** صعوبة متوسطة	**4** صعب جدا	**5** صعب جدا و لا استطيع فعل هذا ابدا	**6** لا أفعل هذا

9) الدخول والخروج من حوض الاستحمام (البانيو):

**1** مش صعب ابدا	**2** صعوبة بسيطة	**3** صعوبة متوسطة	**4** صعب جدا	**5** صعب جدا و لا استطيع فعل هذا ابدا	**6** لا أفعل هذا

10) القيام من السرير:

**1** مش صعب ابدا	**2** صعوبة بسيطة	**3** صعوبة متوسطة	**4** صعب جدا	**5** صعب جدا و لا استطيع فعل هذا ابدا	**6** لا أفعل هذا

.

11) القيام من علي الكرسي:

**1** مش صعب ابدا	**2** صعوبة بسيطة	**3** صعوبة متوسطة	**4** صعب جدا	**5** صعب جدا و لا استطيع فعل هذا ابدا	**6** لا أفعل هذا

12) تتني ركبتيك:

**1** مش صعب ابدا	**2**صعوبة بسيطة	**3** صعوبة متوسطة	**4** صعب جدا	**5** صعب جدا و لا استطيع فعل هذا ابدا	**6** لا أفعل هذا

13) تقدر تشيل حاجة من الأرض:

**1** مش صعب ابدا	**2** صعوبة بسيطة	**3** صعوبة متوسطة	**4** صعب جدا	**5** صعب جدا و لا استطيع فعل هذا ابدا	**6** لا أفعل هذا

14) طلوع السلم:

**1** مش صعب ابدا	**2** صعوبة بسيطة	**3** صعوبة متوسطة	**4** صعب جدا	**5** صعب جدا و لا استطيع فعل هذا ابدا	**6** لا أفعل هذا

15) نزول السلم :

**1** مش صعب ابدا	**2** صعوبة بسيطة	**3** صعوبة متوسطة	**4** صعب جدا	**5** صعب جدا و لا استطيع فعل هذا ابدا	**6** لا أفعل هذا

.

16) ركوب الدراجة او العجلة:

**1** مش صعب ابدا	**2** صعوبة بسيطة	**3** صعوبة متوسطة	**4** صعب جدا	**5** صعب جدا و لا استطيع فعل هذا ابدا	**6** لا أفعل هذا

17) المشي في منزلك او بيتك :

**1** مش صعب ابدا	**2** صعوبة بسيطة	**3** صعوبة متوسطة	**4** صعب جدا	**5** صعب جدا و لا استطيع فعل هذا ابدا	**6** لا أفعل هذا

18) المشي بالخارج او بره البيت:

**1** مش صعب ابدا	**2** صعوبة بسيطة	**3** صعوبة متوسطة	**4** صعب جدا	**5** صعب جدا و لا استطيع فعل هذا ابدا	**6** لا أفعل هذا

19) طلوع أو نزول مرتفعات مثل شوارع مش مستوية او تلال:

**1** مش صعب ابدا	**2** صعوبة بسيطة	**3** صعوبة متوسطة	**4** صعب جدا	**5** صعب جدا و لا استطيع فعل هذا ابدا	**6** لا أفعل هذا

20) الوقوف بشكل مستقيم (مفرود):

**1** مش صعب ابدا	**2** صعوبة بسيطة	**3** صعوبة متوسطة	**4** صعب جدا	**5** صعب جدا و لا استطيع فعل هذا ابدا	**6** لا أفعل هذا

21) إذا سندت علي ركبك، تقدر تقوم تقف؟:

**1** مش صعب ابدا	**2** صعوبة بسيطة	**3** صعوبة متوسطة	**4** صعب جدا	**5** صعب جدا و لا استطيع فعل هذا ابدا	**6** لا أفعل هذا

22) الدخول والخروج من السيارة هو:

**1** مش صعب ابدا	**2** صعوبة بسيطة	**3** صعوبة متوسطة	**4** صعب جدا	**5** صعب جدا و لا استطيع فعل هذا ابدا	**6** لا أفعل هذا

23) المشاركة في الأنشطة المدرسية هي:

**1** مش صعب ابدا	**2** صعوبة بسيطة	**3** صعوبة متوسطة	**4** صعب جدا	**5** صعب جدا و لا استطيع فعل هذا ابدا	**6** لا أفعل هذا

24) الذهاب إلى المدرسة كل يوم ، لآخر اليوم هو:

**1** مش صعب ابدا	**2** صعوبة بسيطة	**3** صعوبة متوسطة	**4** صعب جدا	**5** صعب جدا و لا استطيع فعل هذا ابدا	**6** لا أفعل هذا

25) لعب ألعابك المفضلة هو:

**1** مش صعب ابدا	**2** صعوبة بسيطة	**3** صعوبة متوسطة	**4** صعب جدا	**5** صعب جدا و لا استطيع فعل هذا ابدا	**6** لا أفعل هذا

26) اللعب مع الأصدقاء والعائلة هو:

**1** مش صعب ابدا	**2** صعوبة بسيطة	**3** صعوبة متوسطة	**4** صعب جدا	**5** صعب جدا و لا استطيع فعل هذا ابدا	**6** لا أفعل هذا

27) ممارسة الرياضة اللي متعود عليها :

**1** مش صعب ابدا	**2** صعوبة بسيطة	**3** صعوبة متوسطة	**4** صعب جدا	**5** صعب جدا و لا استطيع فعل هذا ابدا	**6** لا أفعل هذا

28) الجري:

**1** مش صعب ابدا	**2** صعوبة بسيطة	**3** صعوبة متوسطة	**4** صعب جدا	**5** صعب جدا و لا استطيع فعل هذا ابدا	**6** لا أفعل هذا

29) تقدر تكمل لعب او تمارس الانشطة زي اصحابك (المواكبة مع الأصدقاء):

**1** مش صعب ابدا	**2** صعوبة بسيطة	**3** صعوبة متوسطة	**4** صعب جدا	**5** صعب جدا و لا استطيع فعل هذا ابدا	**6** لا أفعل هذا

30) المشي لمدة طويلة (أكثر من ساعة) هو:

**1** مش صعب ابدا	**2** صعوبة بسيطة	**3** صعوبة متوسطة	**4** صعب جدا	**5** صعب جدا و لا استطيع فعل هذا ابدا	**6** لا أفعل هذا

<u>أسئلة عن المشاعر</u>

31) عندك إحساس بالقلق او الخوف من اللي ممكن يحصل؟

**1** فى كل الأوقات	**2** فى معظم الأوقات	**3** فى كثير من الأوقات	**4** فى بعض الأوقات	**5** فى قليل من الأوقات	**6** لا أشعر فى أى من الأوقات

32) عندك احساس بالحزن و إن مافيش حاجة ممكن تفرحك ؟

**1** فى كل الأوقات	**2** فى معظم الأوقات	**3** فى كثير من الأوقات	**4** فى بعض الأوقات	**5** فى قليل من الأوقات	**6** لا أشعر فى أى من الأوقات

33) عندك إحساس بالتعب و انك مش عايز تلعب او تشارك في اي حاجة؟

**1** فى كل الأوقات	**2** فى معظم الأوقات	**3** فى كثير من الأوقات	**4** فى بعض الأوقات	**5** فى قليل من الأوقات	**6** لا أشعر فى أى من الأوقات

34) عندك مشكلة تركز في المدرسة او فى وقت المذاكرة والواجب؟

**1** فى كل الأوقات	**2** فى معظم الأوقات	**3** فى كثير من الأوقات	**4** فى بعض الأوقات	**5** فى قليل من الأوقات	**6** لا أشعر فى أى من الأوقات

35) هل تشعر بسهولة ان مزاجك سئ؟

**1** فى كل الأوقات	**2** فى معظم الأوقات	**3** فى كثير من الأوقات	**4** فى بعض الأوقات	**5** فى قليل من الأوقات	**6** لا أشعر فى أى من الأوقات

36) هل تشعر انك عايز تزعق او تكسر حاجة عندما تكون مضطر للانتظار أو مايحصلش اللى انت عايزه؟

**1** فى كل الأوقات	**2** فى معظم الأوقات	**3** فى كثير من الأوقات	**4** فى بعض الأوقات	**5** فى قليل من الأوقات	**6** لا أشعر فى أى من الأوقات

<u>عمل</u> كل الأشياء التي أريد أن أفعلها هي:

(الرجاء وضع علامة على السطر التالى)

10 صعب جدا ———————— 0 سهل

كيف تشعر اتجاه ما يمكنك فعله ؟:

(الرجاء وضع علامة على السطر التالى)

10 جيد جدا ———————— 0 سيئ جدا

هل نسينا نسألك عن شيء تاني صعب تعمله؟

اطبعها او اكتبها على هذه الخطوط:

____

____

____

ايه اكتر حاجة ضايقتك بالنسبة للعملية؟

____

____

____

أرجوك اتاكد انك جاوبت كل الأسالة

شكرا علي وقتك و اجابتك للاسئلة

الإصدار: ديسمبر 2021

PAEDIATRIC TESS (pTESS-Arm)
ARM QUESTIONNAIRE

استبيان ما بعد جراحات انقاذ الأطراف
استبيان تورونتو المعدل للأطفال
استبيان للذراع

في الاسئلة اللي جاية هنسالك علي بعض الاشياء بتعملها في الحياة و عايزين نعرف اذا كان سهل عليك انك تعملها الاسبوع اللي فات. ممكن تكون في حاجات مقدرتش تعملها لكن مفيش مشكلة. عايزينك تختار الاجابة اللي تحس انها صح بالنسبة لك.

لو سمحت لا تطلب من والديك مساعدتك في الإجابات. لو محتاج مساعدة في قراءة كلمة ، ممكن تطلب منهم قراءتها لك ، ولكن لازم تجاوب بنفسك.

مثال:

استخدام القلم

**1** مش صعب ابدا	**2** صعوبة بسيطة	**3** صعوبة متوسطة	**4** صعب جدا	**5** صعب جدا و لا استطيع فعل هذا ابدا	**6** لا أفعل هذا

لو في حاجة كنت متعود تعملها لكن بسبب ذراعك مش قادر تعملها دلوقتي اختار الاجابة رقم "5 "صعب جدا. لا أستطيع أن أفعل هذا"

لو مش متعود تعمل النشاط ده ، اختار الإجابة رقم "6".

1) لبس البنطلون:

**1** مش صعب ابدا	**2** صعوبة بسيطة	**3** صعوبة متوسطة	**4** صعب جدا	**5** صعب جدا و لا استطيع فعل هذا ابدا	**6** لا أفعل هذا

2) ربط رباط الحذاء:

**1** مش صعب ابدا	**2** صعوبة بسيطة	**3** صعوبة متوسطة	**4** صعب جدا	**5** صعب جدا و لا استطيع فعل هذا ابدا	**6** لا أفعل هذا

3) لبس الشراب:

**1** مش صعب ابدا	**2** صعوبة بسيطة	**3** صعوبة متوسطة	**4** صعب جدا	**5** صعب جدا و لا استطيع فعل هذا ابدا	**6** لا أفعل هذا

4) الاستحمام من غير مساعدة احد:

**1** مش صعب ابدا	**2** صعوبة بسيطة	**3** صعوبة متوسطة	**4** صعب جدا	**5** صعب جدا و لا استطيع فعل هذا ابدا	**6** لا أفعل هذا

5) لبس الجاكت :

**1** مش صعب ابدا	**2** صعوبة بسيطة	**3** صعوبة متوسطة	**4** صعب جدا	**5** صعب جدا و لا استطيع فعل هذا ابدا	**6** لا أفعل هذا

6) قفل زراير القميص:

**1** مش صعب ابدا	**2** صعوبة بسيطة	**3** صعوبة متوسطة	**4** صعب جدا	**5** صعب جدا و لا استطيع فعل هذا ابدا	**6** لا أفعل هذا

7) الطباعة او الكتابة:

**1** مش صعب ابدا	**2** صعوبة بسيطة	**3** صعوبة متوسطة	**4** صعب جدا	**5** صعب جدا و لا استطيع فعل هذا ابدا	**6** لا أفعل هذا

8) تلوين صورة:

**1** مش صعب ابدا	**2** صعوبة بسيطة	**3** صعوبة متوسطة	**4** صعب جدا	**5** صعب جدا و لا استطيع فعل هذا ابدا	**6** لا أفعل هذا

9) غسل اسنانك:

**1** مش صعب ابدا	**2** صعوبة بسيطة	**3** صعوبة متوسطة	**4** صعب جدا	**5** صعب جدا و لا استطيع فعل هذا ابدا	**6** لا أفعل هذا

10) استخدام فرشاة الشعر :

**1** مش صعب ابدا	**2** صعوبة بسيطة	**3** صعوبة متوسطة	**4** صعب جدا	**5** صعب جدا و لا استطيع فعل هذا ابدا	**6** لا أفعل هذا

11) تنظيف غرفتك:

**1** مش صعب ابدا	**2** صعوبة بسيطة	**3** صعوبة متوسطة	**4** صعب جدا	**5** صعب جدا و لا استطيع فعل هذا ابدا	**6** لا أفعل هذا

12) تقطيع او تقشير الفاكهة:

**1** مش صعب ابدا	**2** صعوبة بسيطة	**3** صعوبة متوسطة	**4** صعب جدا	**5** صعب جدا و لا استطيع فعل هذا ابدا	**6** لا أفعل هذا

13) تقطيع الطعام(مثل الدجاج) اثناء الاكل :

**1** مش صعب ابدا	**2** صعوبة بسيطة	**3** صعوبة متوسطة	**4** صعب جدا	**5** صعب جدا و لا استطيع فعل هذا ابدا	**6** لا أفعل هذا

14) الشرب من كوب:

**1** مش صعب ابدا	**2** صعوبة بسيطة	**3** صعوبة متوسطة	**4** صعب جدا	**5** صعب جدا و لا استطيع فعل هذا ابدا	**6** لا أفعل هذا

15) حمل اشياء ثقيلة:

**1** مش صعب ابدا	**2** صعوبة بسيطة	**3** صعوبة متوسطة	**4** صعب جدا	**5** صعب جدا و لا استطيع فعل هذا ابدا	**6** لا أفعل هذا

16) المساعدة في التسوق:

**1** مش صعب ابدا	**2** صعوبة بسيطة	**3** صعوبة متوسطة	**4** صعب جدا	**5** صعب جدا و لا استطيع فعل هذا ابدا	**6** لا أفعل هذا

17) مد اليدين لاعطاء او الحصول علي اموال:

**1** مش صعب ابدا	**2** صعوبة بسيطة	**3** صعوبة متوسطة	**4** صعب جدا	**5** صعب جدا و لا استطيع فعل هذا ابدا	**6** لا أفعل هذا

18) حمل حقيبة علي الظهر:

**1** مش صعب ابدا	**2** صعوبة بسيطة	**3** صعوبة متوسطة	**4** صعب جدا	**5** صعب جدا و لا استطيع فعل هذا ابدا	**6** لا أفعل هذا

19) رفع صندوق و وضعة علي رف عالي:

**1** مش صعب ابدا	**2** صعوبة بسيطة	**3** صعوبة متوسطة	**4** صعب جدا	**5** صعب جدا و لا استطيع فعل هذا ابدا	**6** لا أفعل هذا

20) وضع مفتاح في القفل و لفه:

**1** مش صعب ابدا	**2** صعوبة بسيطة	**3** صعوبة متوسطة	**4** صعب جدا	**5** صعب جدا و لا استطيع فعل هذا ابدا	**6** لا أفعل هذا

21) شد او دفع الباب لفتحة:

**1** مش صعب ابدا	**2** صعوبة بسيطة	**3** صعوبة متوسطة	**4** صعب جدا	**5** صعب جدا و لا استطيع فعل هذا ابدا	**6** لا أفعل هذا

22) التقاط الاشياء الصغيرة:

**1** مش صعب ابدا	**2** صعوبة بسيطة	**3** صعوبة متوسطة	**4** صعب جدا	**5** صعب جدا و لا استطيع فعل هذا ابدا	**6** لا أفعل هذا

23) **عمل الواجب المدرسي:**

**1** مش صعب ابدا	**2** صعوبة بسيطة	**3** صعوبة متوسطة	**4** صعب جدا	**5** صعب جدا و لا استطيع فعل هذا ابدا	**6** لا أفعل هذا

24) **الذهاب للمدرسة كل يوم:**

**1** مش صعب ابدا	**2** صعوبة بسيطة	**3** صعوبة متوسطة	**4** صعب جدا	**5** صعب جدا و لا استطيع فعل هذا ابدا	**6** لا أفعل هذا

25) **اللعب بالعابك المفضلة:**

**1** مش صعب ابدا	**2** صعوبة بسيطة	**3** صعوبة متوسطة	**4** صعب جدا	**5** صعب جدا و لا استطيع فعل هذا ابدا	**6** لا أفعل هذا

26) **اللعب مع الاصدقاء والعائلة:**

**1** مش صعب ابدا	**2** صعوبة بسيطة	**3** صعوبة متوسطة	**4** صعب جدا	**5** صعب جدا و لا استطيع فعل هذا ابدا	**6** لا أفعل هذا

27) **ممارسة الرياضة المفضلة:**

**1** مش صعب ابدا	**2** صعوبة بسيطة	**3** صعوبة متوسطة	**4** صعب جدا	**5** صعب جدا و لا استطيع فعل هذا ابدا	**6** لا أفعل هذا

أسئلة عن المشاعر

28) **عندك إحساس بالقلق او الخوف من اللي ممكن يحصل؟**

**1** فى كل الأوقات	**2** فى معظم الأوقات	**3** فى كثير من الأوقات	**4** فى بعض الأوقات	**5** فى قليل من الأوقات	**6** لا أشعر فى أى من الأوقات

29) **عندك احساس بالحزن و إن مافيش حاجة ممكن تفرحك ؟**

**1** فى كل الأوقات	**2** فى معظم الأوقات	**3** فى كثير من الأوقات	**4** فى بعض الأوقات	**5** فى قليل من الأوقات	**6** لا أشعر فى أى من الأوقات

30) عندك إحساس بالتعب و انك مش عايز تلعب او تشارك في اي حاجة؟

**1** فى كل الأوقات	**2** فى معظم الأوقات	**3** فى كثير من الأوقات	**4** فى بعض الأوقات	**5** فى قليل من الأوقات	**6** لا أشعر فى أى من الأوقات

31) عندك مشكلة تركز في المدرسة او فى وقت المذاكرة والواجب؟

**1** فى كل الأوقات	**2** فى معظم الأوقات	**3** فى كثير من الأوقات	**4** فى بعض الأوقات	**5** فى قليل من الأوقات	**6** لا أشعر فى أى من الأوقات

32) هل تشعر بسهولة ان مزاجك سئ؟

**1** فى كل الأوقات	**2** فى معظم الأوقات	**3** فى كثير من الأوقات	**4** فى بعض الأوقات	**5** فى قليل من الأوقات	**6** لا أشعر فى أى من الأوقات

33) هل تشعر انك عايز تزعق او تكسر حاجة عندما تكون مضطر للانتظار أو مايحصلش اللى انت عايزه؟

**1** فى كل الأوقات	**2** فى معظم الأوقات	**3** فى كثير من الأوقات	**4** فى بعض الأوقات	**5** فى قليل من الأوقات	**6** لا أشعر فى أى من الأوقات

عمل كل الأشياء التي أريد أن أفعلها هي:

(الرجاء وضع علامة على السطر التالى)

0 ———————— 10

سهل ... صعب جدا جدا

كيف تشعر اتجاه ما يمكنك فعله ؟:

(الرجاء وضع علامة على السطر التالى)

0 ———————— 10

سيئ جدا ... جيد جدا

هل نسينا نسألك عن شيء تاني صعب تعمله؟
اطبعها او اكتبها على هذه الخطوط:

ايه اكتر حاجة ضايقتك بالنسبة للعملية؟

أرجوك اتاكد انك جاوبت كل الأسالة
شكرا علي وقتك و اجابتك للاسئلة

استبيان ما بعد جراحات انقاذ الأطراف
استبيان تورونتو
استبيان للساق
Toronto Extremity Salvage Score
(TESS-Leg)

في الاسئلة اللي جاية هنسالك علي بعض الاشياء بتعملها في الحياة و عايزين نعرف اذا كان سهل عليك انك تعملها الاسبوع اللي فات. ممكن تكون في حاجات مقدرتش تعملها لكن مفيش مشكلة. عايزينك تختار الاجابة اللي تحس انها صح بالنسبة لك.

لو سمحت لا تطلب من والديك مساعدتك في الإجابات. لو محتاج مساعدة في قراءة كلمة ، ممكن تطلب منهم قراءتها لك ، ولكن لازم تجاوب بنفسك.

مثال:

ركوب العجلة :

**1** مش صعب ابدا	**2** صعوبة بسيطة	**3** صعوبة متوسطة	**4** صعب جدا	**5** صعب جدا و لا استطيع فعل هذا ابدا	**6** لا أفعل هذا

لو في حاجة كنت متعود تعملها لكن بسبب رجلك مش قادر تعملها دلوقتي اختار الاجابة رقم "5 "صعب جدا. لا أستطيع أن أفعل هذا" لو مش متعود تعمل النشاط ده ، اختار الإجابة "6".

الإصدار: ديسمبر 2021

عايزين نعرف اذا كان من السهل عليك القيام بالأنشطة التالية الأسبوع الي فات:

1) لبس البنطالون :

**1** مش صعب ابدا	**2** صعوبة بسيطة	**3** صعوبة متوسطة	**4** صعب جدا	**5** صعب جدا و لا استطيع فعل هذا ابدا	**6** لا أفعل هذا

2) لبس الحذاء:

**1** مش صعب ابدا	**2** صعوبة بسيطة	**3** صعوبة متوسطة	**4** صعب جدا	**5** صعب جدا و لا استطيع فعل هذا ابدا	**6** لا أفعل هذا

3) لبس الشراب:

**1** مش صعب ابدا	**2** صعوبة بسيطة	**3** صعوبة متوسطة	**4** صعب جدا	**5** صعب جدا و لا استطيع فعل هذا ابدا	**6** لا أفعل هذا

4) الاستحمام من غير مساعدة احد:

**1** مش صعب ابدا	**2** صعوبة بسيطة	**3** صعوبة متوسطة	**4** صعب جدا	**5** صعب جدا و لا استطيع فعل هذا ابدا	**6** لا أفعل هذا

5) القيام بالأعمال المنزلية الخفيفة مثل التلميع و الترتيب:

**1** مش صعب ابدا	**2** صعوبة بسيطة	**3** صعوبة متوسطة	**4** صعب جدا	**5** صعب جدا و لا استطيع فعل هذا ابدا	**6** لا أفعل هذا

6) القيام بأى نشاط زراعى:

**1** مش صعب ابدا	**2** صعوبة بسيطة	**3** صعوبة متوسطة	**4** صعب جدا	**5** صعب جدا و لا استطيع فعل هذا ابدا	**6** لا أفعل هذا

7) تحضير وتقديم وجبات الطعام

**1** مش صعب ابدا	**2** صعوبة بسيطة	**3** صعوبة متوسطة	**4** صعب جدا	**5** صعب جدا و لا استطيع فعل هذا ابدا	**6** لا أفعل هذا

الإصدار: ديسمبر 2021

8) الذهاب للتسوق:

**1** مش صعب ابدا	**2** صعوبة بسيطة	**3** صعوبة متوسطة	**4** صعب جدا	**5** صعب جدا و لا استطيع فعل هذا ابدا	**6** لا أفعل هذا

9) القيام بالأعمال المنزلية الشاقة مثل تحريك أثاث/ أشياء ثقيلة أو الكنس:

**1** مش صعب ابدا	**2** صعوبة بسيطة	**3** صعوبة متوسطة	**4** صعب جدا	**5** صعب جدا و لا استطيع فعل هذا ابدا	**6** لا أفعل هذا

10) الدخول والخروج من حوض الاستحمام (البانيو):

**1** مش صعب ابدا	**2** صعوبة بسيطة	**3** صعوبة متوسطة	**4** صعب جدا	**5** صعب جدا و لا استطيع فعل هذا ابدا	**6** لا أفعل هذا

11) القيام من السرير:

**1** مش صعب ابدا	**2** صعوبة بسيطة	**3** صعوبة متوسطة	**4** صعب جدا	**5** صعب جدا و لا استطيع فعل هذا ابدا	**6** لا أفعل هذا

.

12) القيام من علي الكرسي:

**1** مش صعب ابدا	**2** صعوبة بسيطة	**3** صعوبة متوسطة	**4** صعب جدا	**5** صعب جدا و لا استطيع فعل هذا ابدا	**6** لا أفعل هذا

13) تتني ركبتيك أو تركع:

**1** مش صعب ابدا	**2**صعوبة بسيطة	**3** صعوبة متوسطة	**4** صعب جدا	**5** صعب جدا و لا استطيع فعل هذا ابدا	**6** لا أفعل هذا

14) تقدر تشيل حاجة من الأرض:

**1** مش صعب ابدا	**2** صعوبة بسيطة	**3** صعوبة متوسطة	**4** صعب جدا	**5** صعب جدا و لا استطيع فعل هذا ابدا	**6** لا أفعل هذا

15) طلوع السلم:

**1** مش صعب ابدا	**2** صعوبة بسيطة	**3** صعوبة متوسطة	**4** صعب جدا	**5** صعب جدا و لا استطيع فعل هذا ابدا	**6** لا أفعل هذا

16) نزول السلم:

**1** مش صعب ابدا	**2** صعوبة بسيطة	**3** صعوبة متوسطة	**4** صعب جدا	**5** صعب جدا و لا استطيع فعل هذا ابدا	**6** لا أفعل هذا

17) قيادة السيارة:

**1** مش صعب ابدا	**2** صعوبة بسيطة	**3** صعوبة متوسطة	**4** صعب جدا	**5** صعب جدا و لا استطيع فعل هذا ابدا	**6** لا أفعل هذا

18) المشي في منزلك او بيتك :

**1** مش صعب ابدا	**2** صعوبة بسيطة	**3** صعوبة متوسطة	**4** صعب جدا	**5** صعب جدا و لا استطيع فعل هذا ابدا	**6** لا أفعل هذا

19) المشي بالخارج او بره البيت:

**1** مش صعب ابدا	**2** صعوبة بسيطة	**3** صعوبة متوسطة	**4** صعب جدا	**5** صعب جدا و لا استطيع فعل هذا ابدا	**6** لا أفعل هذا

20) الجلوس:

**1** مش صعب ابدا	**2** صعوبة بسيطة	**3** صعوبة متوسطة	**4** صعب جدا	**5** صعب جدا و لا استطيع فعل هذا ابدا	**6** لا أفعل هذا

21) طلوع أو نزول مرتفعات مثل شوارع مش مستوية او تلال:

**1** مش صعب ابدا	**2** صعوبة بسيطة	**3** صعوبة متوسطة	**4** صعب جدا	**5** صعب جدا و لا استطيع فعل هذا ابدا	**6** لا أفعل هذا

22) الوقوف بشكل مستقيم (مفرود):

**1** مش صعب ابدا	**2** صعوبة بسيطة	**3** صعوبة متوسطة	**4** صعب جدا	**5** صعب جدا و لا استطيع فعل هذا ابدا	**6** لا أفعل هذا

23) إذا سندت علي ركبك، تقدر تقوم تقف؟:

**1** مش صعب ابدا	**2** صعوبة بسيطة	**3** صعوبة متوسطة	**4** صعب جدا	**5** صعب جدا و لا استطيع فعل هذا ابدا	**6** لا أفعل هذا

24) الدخول والخروج من السيارة هو:

**1** مش صعب ابدا	**2** صعوبة بسيطة	**3** صعوبة متوسطة	**4** صعب جدا	**5** صعب جدا و لا استطيع فعل هذا ابدا	**6** لا أفعل هذا

25) علاقة زوجبة/ حميمة:

**1** مش صعب ابدا	**2** صعوبة بسيطة	**3** صعوبة متوسطة	**4** صعب جدا	**5** صعب جدا و لا استطيع فعل هذا ابدا	**6** لا أفعل هذا

26) إتمام واجباتي المعتادة في الدراسة أو فى العمل (يشمل العمل وظيفة خارج المنزل أو ربة منزل):

**1** مش صعب ابدا	**2** صعوبة بسيطة	**3** صعوبة متوسطة	**4** صعب جدا	**5** صعب جدا و لا استطيع فعل هذا ابدا	**6** لا أفعل هذا

27) أعمل نفس عدد ساعات زملائى بالعمل (يشمل العمل وظيفة خارج المنزل أو ربة منزل) **أو أكمل اليوم الدراسى لأخره:**

**1** مش صعب ابدا	**2** صعوبة بسيطة	**3** صعوبة متوسطة	**4** صعب جدا	**5** صعب جدا و لا استطيع فعل هذا ابدا	**6** لا أفعل هذا

28) المشاركة في الأنشطة الترفيهية المعتادة:

**1** مش صعب ابدا	**2** صعوبة بسيطة	**3** صعوبة متوسطة	**4** صعب جدا	**5** صعب جدا و لا استطيع فعل هذا ابدا	**6** لا أفعل هذا

29) التواصل الاجتماعى مع الأصدقاء والعائلة (حضور تجمعات و احتفالات):

**1** مش صعب ابدا	**2** صعوبة بسيطة	**3** صعوبة متوسطة	**4** صعب جدا	**5** صعب جدا و لا استطيع فعل هذا ابدا	**6** لا أفعل هذا

30) ممارسة الرياضة اللي متعود عليها:

**1** مش صعب ابدا	**2** صعوبة بسيطة	**3** صعوبة متوسطة	**4** صعب جدا	**5** صعب جدا و لا استطيع فعل هذا ابدا	**6** لا أفعل هذا

أسئلة عن المشاعر

31) عندك إحساس بالقلق او الخوف من اللي ممكن يحصل؟

**1** فى كل الأوقات	**2** فى معظم الأوقات	**3** فى كثير من الأوقات	**4** فى بعض الأوقات	**5** فى قليل من الأوقات	**6** لا أشعر فى أى من الأوقات

32) عندك احساس بالحزن و إن مافيش حاجة ممكن تفرحك ؟

**1** فى كل الأوقات	**2** فى معظم الأوقات	**3** فى كثير من الأوقات	**4** فى بعض الأوقات	**5** فى قليل من الأوقات	**6** لا أشعر فى أى من الأوقات

33) عندك إحساس بالتعب و انك مش عايز تشارك في اي حاجة؟

**1** فى كل الأوقات	**2** فى معظم الأوقات	**3** فى كثير من الأوقات	**4** فى بعض الأوقات	**5** فى قليل من الأوقات	**6** لا أشعر فى أى من الأوقات

34) عندك مشكلة تركز في الشغل أو الدراسة؟

**1** فى كل الأوقات	**2** فى معظم الأوقات	**3** فى كثير من الأوقات	**4** فى بعض الأوقات	**5** فى قليل من الأوقات	**6** لا أشعر فى أى من الأوقات

35) هل تشعر بسهولة ان مزاجك سئ؟

**1** فى كل الأوقات	**2** فى معظم الأوقات	**3** فى كثير من الأوقات	**4** فى بعض الأوقات	**5** فى قليل من الأوقات	**6** لا أشعر فى أى من الأوقات

36) هل تشعر انك عايز تزعق او تكسر حاجة عندما تكون مضطر للانتظار أو مايحصلش اللى انت عايزه؟

**1** فى كل الأوقات	**2** فى معظم الأوقات	**3** فى كثير من الأوقات	**4** فى بعض الأوقات	**5** فى قليل من الأوقات	**6** لا أشعر فى أى من الأوقات

بشكل عام، عمل كل الأشياء التي أريد أن أفعلها هي:

**1** مش صعب ابدا	**2** صعوبة بسيطة	**3** صعوبة متوسطة	**4** صعب جدا	**5** صعب جدا و لا استطيع فعلها

بشكل عام، كيف تشعر اتجاه ما يمكنك فعله (مستوى قدرتك) ؟:

**1** قادر تماما	**2** عدم قدرة بسيطة	**3** عدم قدرة متوسطة	**4** عدم قدرة شديدة	**5** غير قادر تماما

هل نسينا نسألك عن شيء تاني صعب تعمله؟
اطبعها او اكتبها على هذه الخطوط:

____

____

____

ايه اكتر حاجة ضايقتك أو صعوبات واجهتك بسبب العملية؟

____

____

____

أرجوك اتاكد انك جاوبت كل الأسالة
شكرا علي وقتك و اجابتك للاسئلة

الإصدار: ديسمبر 2021

TESS (TESS-Arm)
ARM QUESTIONNAIRE

<u>استبيان ما بعد جراحات انقاذ الأطراف</u>

<u>استبيان تورونتو للذراع</u>

في الاسئلة اللي جاية هنسالك علي بعض الاشياء بتعملها في الحياة و عايزين نعرف اذا كان سهل عليك انك تعملها الاسبوع اللي فات. ممكن تكون في حاجات مقدرتش تعملها لكن مفيش مشكلة. عايزينك تختار الاجابة اللي تحس انها صح بالنسبة لك.

لو سمحت لا تطلب من أحد مساعدتك في الإجابات. لو محتاج مساعدة في قراءة كلمة او فهم سؤال، ممكن تسأل ، ولكن لازم تجاوب بنفسك.

<u>مثال:</u>

تقشير الخضار أو الفاكهة

**1** مش صعب ابدا	**2** صعوبة بسيطة	**3** صعوبة متوسطة	**4** صعب جدا	**5** صعب جدا و لا استطيع فعل هذا ابدا	**6** لا أفعل هذا

لو في حاجة بسبب ذراعك مش قادر تعملها دلوقتي اختار الاجابة رقم "5 "صعب جدا. لا أستطيع أن أفعل هذا"
لو مش متعود تعمل النشاط ده أو مش مهتم تجربه، اختار الإجابة رقم "6".

1) لبس البنطلون:

**1** مش صعب ابدا	**2** صعوبة بسيطة	**3** صعوبة متوسطة	**4** صعب جدا	**5** صعب جدا و لا استطيع فعل هذا ابدا	**6** لا أفعل هذا

2) ربط رباط الحذاء:

**1** مش صعب ابدا	**2** صعوبة بسيطة	**3** صعوبة متوسطة	**4** صعب جدا	**5** صعب جدا و لا استطيع فعل هذا ابدا	**6** لا أفعل هذا

3) لبس الشراب:

**1** مش صعب ابدا	**2** صعوبة بسيطة	**3** صعوبة متوسطة	**4** صعب جدا	**5** صعب جدا و لا استطيع فعل هذا ابدا	**6** لا أفعل هذا

4) الاستحمام من غير مساعدة احد:

**1** مش صعب ابدا	**2** صعوبة بسيطة	**3** صعوبة متوسطة	**4** صعب جدا	**5** صعب جدا و لا استطيع فعل هذا ابدا	**6** لا أفعل هذا

5) لبس تيشرت/بلوفر/جاكت:

**1** مش صعب ابدا	**2** صعوبة بسيطة	**3** صعوبة متوسطة	**4** صعب جدا	**5** صعب جدا و لا استطيع فعل هذا ابدا	**6** لا أفعل هذا

6) قفل زراير القميص:

**1** مش صعب ابدا	**2** صعوبة بسيطة	**3** صعوبة متوسطة	**4** صعب جدا	**5** صعب جدا و لا استطيع فعل هذا ابدا	**6** لا أفعل هذا

7) ربط ربطة عنق (كرافتة) أو فيونكة على رقبة البلوزة:

**1** مش صعب ابدا	**2** صعوبة بسيطة	**3** صعوبة متوسطة	**4** صعب جدا	**5** صعب جدا و لا استطيع فعل هذا ابدا	**6** لا أفعل هذا

8) وضع المكياج أو الحلاقة:

**1** مش صعب ابدا	**2** صعوبة بسيطة	**3** صعوبة متوسطة	**4** صعب جدا	**5** صعب جدا و لا استطيع فعل هذا ابدا	**6** لا أفعل هذا

9) غسل اسنانك:

**1** مش صعب ابدا	**2** صعوبة بسيطة	**3** صعوبة متوسطة	**4** صعب جدا	**5** صعب جدا و لا استطيع فعل هذا ابدا	**6** لا أفعل هذا

10) استخدام فرشاة الشعر:

**1** مش صعب ابدا	**2** صعوبة بسيطة	**3** صعوبة متوسطة	**4** صعب جدا	**5** صعب جدا و لا استطيع فعل هذا ابدا	**6** لا أفعل هذا

11) القيام بالأعمال المنزلية الخفيفة مثل الترتيب أوالتلميع:

**1** مش صعب ابدا	**2** صعوبة بسيطة	**3** صعوبة متوسطة	**4** صعب جدا	**5** صعب جدا و لا استطيع فعل هذا ابدا	**6** لا أفعل هذا

12) القيام بأى نشاط زراعى:

**1** مش صعب ابدا	**2** صعوبة بسيطة	**3** صعوبة متوسطة	**4** صعب جدا	**5** صعب جدا و لا استطيع فعل هذا ابدا	**6** لا أفعل هذا

13) تحضير وتقديم وجبات الطعام:

**1** مش صعب ابدا	**2** صعوبة بسيطة	**3** صعوبة متوسطة	**4** صعب جدا	**5** صعب جدا و لا استطيع فعل هذا ابدا	**6** لا أفعل هذا

14) تقطيع الطعام (مثل الدجاج) اثناء الاكل:

**1** مش صعب ابدا	**2** صعوبة بسيطة	**3** صعوبة متوسطة	**4** صعب جدا	**5** صعب جدا و لا استطيع فعل هذا ابدا	**6** لا أفعل هذا

15) الشرب من كوب:

**1** مش صعب ابدا	**2** صعوبة بسيطة	**3** صعوبة متوسطة	**4** صعب جدا	**5** صعب جدا و لا استطيع فعل هذا ابدا	**6** لا أفعل هذا

16) القيام بالأعمال المنزلية الشاقة مثل تحريك أثاث/ أشياء ثقيلة أو الكنس:

**1** مش صعب ابدا	**2** صعوبة بسيطة	**3** صعوبة متوسطة	**4** صعب جدا	**5** صعب جدا و لا استطيع فعل هذا ابدا	**6** لا أفعل هذا

17) الذهاب للتسوق:

**1** مش صعب ابدا	**2** صعوبة بسيطة	**3** صعوبة متوسطة	**4** صعب جدا	**5** صعب جدا و لا استطيع فعل هذا ابدا	**6** لا أفعل هذا

18) مد اليدين لاعطاء او الحصول علي اموال:

**1** مش صعب ابدا	**2** صعوبة بسيطة	**3** صعوبة متوسطة	**4** صعب جدا	**5** صعب جدا و لا استطيع فعل هذا ابدا	**6** لا أفعل هذا

19) حمل كيس تسوق أو شنطة:

**1** مش صعب ابدا	**2** صعوبة بسيطة	**3** صعوبة متوسطة	**4** صعب جدا	**5** صعب جدا و لا استطيع فعل هذا ابدا	**6** لا أفعل هذا

20) رفع صندوق و وضعة علي رف عالي:

**1** مش صعب ابدا	**2** صعوبة بسيطة	**3** صعوبة متوسطة	**4** صعب جدا	**5** صعب جدا و لا استطيع فعل هذا ابدا	**6** لا أفعل هذا

21) وضع مفتاح في القفل و لفه:

**1** مش صعب ابدا	**2** صعوبة بسيطة	**3** صعوبة متوسطة	**4** صعب جدا	**5** صعب جدا و لا استطيع فعل هذا ابدا	**6** لا أفعل هذا

22) شد او دفع الباب لفتحة:

**1** مش صعب ابدا	**2** صعوبة بسيطة	**3** صعوبة متوسطة	**4** صعب جدا	**5** صعب جدا و لا استطيع فعل هذا ابدا	**6** لا أفعل هذا

23) الكتابة:

**1** مش صعب ابدا	**2** صعوبة بسيطة	**3** صعوبة متوسطة	**4** صعب جدا	**5** صعب جدا و لا استطيع فعل هذا ابدا	**6** لا أفعل هذا

24) التقاط الاشياء الصغيرة:

**1** مش صعب ابدا	**2** صعوبة بسيطة	**3** صعوبة متوسطة	**4** صعب جدا	**5** صعب جدا و لا استطيع فعل هذا ابدا	**6** لا أفعل هذا

25) إتمام واجباتي المعتادة في الدراسة أو العمل (يشمل العمل وظيفة خارج المنزل أو ربة منزل):

**1** مش صعب ابدا	**2** صعوبة بسيطة	**3** صعوبة متوسطة	**4** صعب جدا	**5** صعب جدا و لا استطيع فعل هذا ابدا	**6** لا أفعل هذا

26) أعمل نفس عدد ساعات زملائى بالعمل (يشمل العمل وظيفة خارج المنزل أو ربة منزل) أو أكمل اليوم الدراسى لأخره:

**1** مش صعب ابدا	**2** صعوبة بسيطة	**3** صعوبة متوسطة	**4** صعب جدا	**5** صعب جدا و لا استطيع فعل هذا ابدا	**6** لا أفعل هذا

27) المشاركة في الأنشطة الترفيهية المعتادة:

**1** مش صعب ابدا	**2** صعوبة بسيطة	**3** صعوبة متوسطة	**4** صعب جدا	**5** صعب جدا و لا استطيع فعل هذا ابدا	**6** لا أفعل هذا

28) التواصل الاجتماعى مع الأصدقاء والعائلة (حضور تجمعات و احتفالات):

**1** مش صعب ابدا	**2** صعوبة بسيطة	**3** صعوبة متوسطة	**4** صعب جدا	**5** صعب جدا و لا استطيع فعل هذا ابدا	**6** لا أفعل هذا

29) ممارسة الرياضة اللي متعود عليها:

**1** مش صعب ابدا	**2** صعوبة بسيطة	**3** صعوبة متوسطة	**4** صعب جدا	**5** صعب جدا و لا استطيع فعل هذا ابدا	**6** لا أفعل هذا

أسئلة عن المشاعر

30) عندك إحساس بالقلق او الخوف من اللي ممكن يحصل؟

**1** فى كل الأوقات	**2** فى معظم الأوقات	**3** فى كثير من الأوقات	**4** فى بعض الأوقات	**5** فى قليل من الأوقات	**6** لا أشعر فى أى من الأوقات

31) عندك احساس بالحزن و إن مافيش حاجة ممكن تفرحك ؟

**1** فى كل الأوقات	**2** فى معظم الأوقات	**3** فى كثير من الأوقات	**4** فى بعض الأوقات	**5** فى قليل من الأوقات	**6** لا أشعر فى أى من الأوقات

32) عندك إحساس بالتعب و انك مش عايز تلعب او تشارك في اي حاجة؟

**1** فى كل الأوقات	**2** فى معظم الأوقات	**3** فى كثير من الأوقات	**4** فى بعض الأوقات	**5** فى قليل من الأوقات	**6** لا أشعر فى أى من الأوقات

33) عندك مشكلة تركز في الشغل أو الدراسة؟

**1** فى كل الأوقات	**2** فى معظم الأوقات	**3** فى كثير من الأوقات	**4** فى بعض الأوقات	**5** فى قليل من الأوقات	**6** لا أشعر فى أى من الأوقات

34) هل تشعر بسهولة ان مزاجك سئ؟

**1** فى كل الأوقات	**2** فى معظم الأوقات	**3** فى كثير من الأوقات	**4** فى بعض الأوقات	**5** فى قليل من الأوقات	**6** لا أشعر فى أى من الأوقات

35) هل تشعر انك عايز تزعق او تكسر حاجة عندما تكون مضطر للانتظار أو مايحصلش اللى انت عايزه؟

**1** فى كل الأوقات	**2** فى معظم الأوقات	**3** فى كثير من الأوقات	**4** فى بعض الأوقات	**5** فى قليل من الأوقات	**6** لا أشعر فى أى من الأوقات

بشكل عام، عمل كل الأشياء التي أريد أن أفعلها هي:

**1** مش صعب ابدا	**2** صعوبة بسيطة	**3** صعوبة متوسطة	**4** صعب جدا	**5** صعب جدا و لا استطيع فعلها

بشكل عام، كيف تشعر اتجاه ما يمكنك فعله (مستوى قدرتك) ؟:

**1** قادر تماما	**2** عدم قدرة بسيطة	**3** عدم قدرة متوسطة	**4** عدم قدرة شديدة	**5** غير قادر تماما

هل نسينا نسألك عن شيء تاني صعب تعمله؟
اطبعها او اكتبها على هذه الخطوط:

ايه اكتر حاجة ضايقتك أو صعوبات واجهتك بسبب العملية؟

أرجوك اتاكد انك جاوبت كل الأسالة

شكرا علي وقتك و اجابتك للاسئلة
