# Supplementary material for: Cross-cultural adaptation and validation of a self-reporting tool to assess health-related quality of life for Egyptians with extremity bone sarcomas in childhood or adolescence
Source: Health Qual Life Outcomes. 2023 Jul 29;21:81. doi: 10.1186/s12955-023-02165-3 (PMC10386605; doi:10.1186/s12955-023-02165-3)

Rotated Factor Matrix

	Factor		
	1	2	3
q1	0.44		
q2	0.584		
q3	0.402		
q4	0.567		
q5	0.622		
q6		0.494	
q7	0.538		
q8	0.452		
q9	0.639		
q10	0.71		
q11	0.538		
q12			
q13	0.58		
q14	0.564	0.491	
q15	0.584		
q16		0.651	
q17	0.705		
q18	0.655		
q19	0.515	0.481	
q20			
q21		0.538	
q22	0.611		
q23		0.66	
q24		0.616	
q25		0.592	
q26		0.549	
q27		0.686	
q28		0.696	
q29		0.778	
q30		0.585	
q31			0.584
q32			0.771
q33			0.684
q34			0.476
q35			0.77
q36			0.431

Extraction Method: Principal Axis Factoring.

Rotation Method: Varimax with Kaiser Normalization.

* Rotation converged in 5 iterations.

Factor Plot in Rotated Factor Space

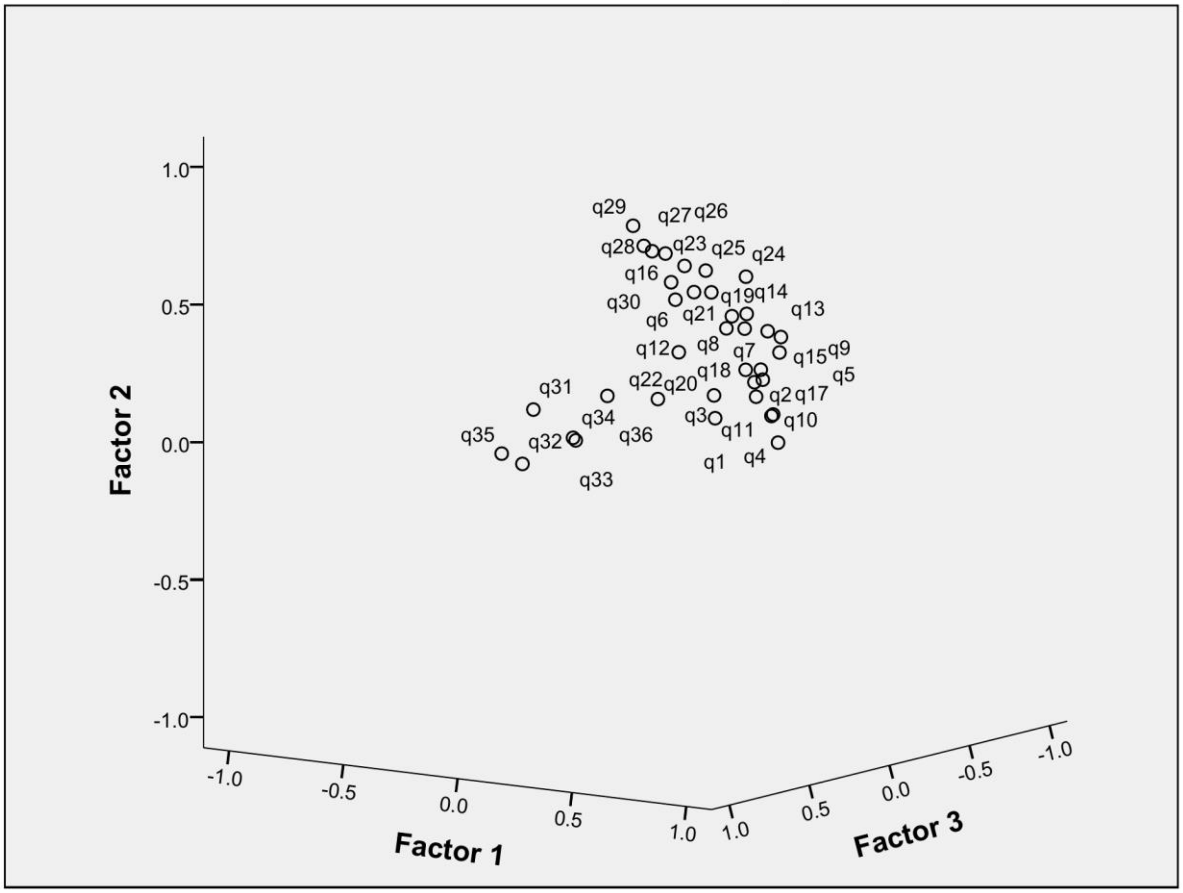

Supplement: Supplementary file 2 — Additional file 2. Rotated factor matrix and plot. [file 12955_2023_2165_MOESM2_ESM.pdf]
